# Supplementary material for: The prevalence of poor sleep quality and associated risk factors among Chinese elderly adults in nursing homes: A cross-sectional study
Source: PLoS One. 2020 May 15;15(5):e0232834. doi: 10.1371/journal.pone.0232834 (PMC7228093; doi:10.1371/journal.pone.0232834)
Supplement: Attachment — Submitted filename: REVIEWER DR EE.docx [file pone.0232834.s001.docx]

PLOS ONE

**The prevalence of poor sleep quality and associated risk factors among Chinese elderly adults in nursing homes: a cross-sectional study**

--Manuscript Draft--

| **Manuscript Number:** | PONE-D-19-31759 |
| --- | --- |
| **Article Type:** | Research Article |
| **Full Title:** | The prevalence of poor sleep quality and associated risk factors among Chinese elderly adults in nursing homes: a cross-sectional study |
| **Short Title:** | poor sleep quality and associated risk factors |
| **Corresponding Author:** | Huilan Xu  Central South University Changsha, CHINA |
| **Keywords:** | sleep quality; nursing home; interaction; risk factors |
| **Abstract:** | Background  Sleep problems have become the most common complaints among elderly adults. There are a few studies indentified prevalence of poor sleep quality and its associated factors in nursing home setting.Therefore, our study aim to examine the prevalence of poor sleep quality, its risk factors, and their interactions among Chinese elderly adults in nursing homes.Methods  A total of 817 elderly residents from 24 nursing homes were included in this cross- sectional study. Sleep quality was assessed using the Pittsburgh Sleep Quality Index (PSQI), and poor sleep quality was defined as PSQI >5. Multiple binary logistic regression was used to estimate adjusted odds ratios (AORs) and 95% confidence intervals (CIs) between risk factors and poor sleep quality. An additional interaction model was used to analyse the interaction between risk factors.Results  The prevalence of poor sleep quality was 67.3% (95% CI: 64.0, 70.5%) among elderly adults in nursing homes. Multiple binary logistic regression results showed that participants with the following characteristics had an increased risk of poor sleep quality after adjustments for other confounders: being 70-79 years old (AOR: 1.81, 95% CI: 1.10, 2.97) or 80 years old and above (AOR: 2.64, 95% CI: 1.67, 4.17); having  less than 7 years of education (AOR: 1.59, 95% CI: 1.08, 2.33); having one to two kinds of chronic diseases (AOR: 2.26, 95% CI: 1.53, 3.32) or three or more kinds of chronic disease (AOR: 2.81, 95% CI: 1.65, 4.76); having depression (AOR: 3.13, 95%  CI: 2.04, 4.81), anxiety (AOR: 3.42, 95% CI: 1.68, 6.97), and lower social support  (AOR: 1.57, 95% CI: 1.11, 2.21). Additive interactions were detected between age and  anxiety (AOR: 8.34, 95% CI: 4.43, 15.69), between chronic disease and anxiety (AOR:  8.61, 95% CI; 4.28, 17.31) and between social support and anxiety (AOR: 6.43, 95%  CI: 3.22, 12.86).Conclusions  The prevalence of poor sleep quality in nursing homes is relatively high. Anxiety has additive interactions with age, chronic disease and social support for poor sleep quality. These findings have significant implications for interventions that aim to improve sleep quality among elderly residents in nursing homes. |
| **Order of Authors:** | Zhao Hu |
|  | Xidi Zhu |
|  | Yunhan Yu |
|  | Huilan Xu |
| **Additional Information:** |  |
| **Question** | **Response** |
| **Financial Disclosure** | The author(s) received no specific funding for this work |

| Enter a financial disclosure statement that describes the sources of funding for the work included in this submission. Review the [submission guidelines](http://journals.plos.org/plosone/s/submission-guidelines#loc-financial-disclosure-statement) for detailed requirements. View published research articles from [*PLOS ONE*](http://journals.plos.org/plosone/) for specific examples.  This statement is required for submission and **will appear in the published article** if the submission is accepted. Please make sure it is accurate. |  |
| --- | --- |
| **Unfunded studies**  Enter: *The author(s) received no specific funding for this work.*  **Funded studies**  Enter a statement with the following details:   - Initials of the authors who received each award - Grant numbers awarded to each author - The full name of each funder - URL of each funder website - Did the sponsors or funders play any role in the study design, data collection and analysis, decision to publish, or preparation of the manuscript? - **NO** - Include this sentence at the end of your statement: *The funders had no role in study design, data collection and analysis, decision to publish, or preparation of the manuscript.* - **YES** - Specify the role(s) played. |  |
| * typeset |  |
| **Competing Interests**  Use the instructions below to enter a competing interest statement for this submission. On behalf of all authors, disclose any [competing interests](http://journals.plos.org/plosone/s/competing-interests) that could be perceived to bias this work—acknowledging all financial support and any other relevant financial or non- financial competing interests.  This statement **will appear in the published article** if the submission is accepted. Please make sure it is accurate. View published research articles | The authors have declared that no competing interests exist. |

| from [*PLOS ONE*](http://journals.plos.org/plosone/) for specific examples. |  |
| --- | --- |
| **NO authors have competing interests**  Enter: *The authors have declared that no competing interests exist.*  **Authors with competing interests**  Enter competing interest details beginning with this statement:  *I have read the journal's policy and the authors of this manuscript have the following competing interests: [insert competing interests here]* |  |
| * typeset |  |
| **Ethics Statement**  Enter an ethics statement for this submission. This statement is required if the study involved:   - Human participants - Human specimens or tissue - Vertebrate animals or cephalopods - Vertebrate embryos or tissues - Field research   Write "N/A" if the submission does not require an ethics statement.  General guidance is provided below. Consult the [submission guidelines](http://journals.plos.org/plosone/s/submission-guidelines#loc-guidelines-for-specific-study-types) for detailed instructions. **Make sure that all information entered here is included in the Methods section of the manuscript.** | This study was approved by the Ethical Committee of Xiangya School of Public Health in Central South University (No.XYGW-2018-49) |

| **Format for specific study types**  **Human Subject Research (involving human participants and/or tissue)**   - Give the name of the institutional review board or ethics committee that approved the study - Include the approval number and/or a statement indicating approval of this research - Indicate the form of consent obtained (written/oral) or the reason that consent was not obtained (e.g. the data were analyzed anonymously)   **Animal Research (involving vertebrate animals, embryos or tissues)**   - Provide the name of the Institutional Animal Care and Use Committee (IACUC) or other relevant ethics board that reviewed the study protocol, and indicate whether they approved this research or granted a formal waiver of ethical approval - Include an approval number if one was obtained - If the study involved *non-human primates*, add *additional details* about animal welfare and steps taken to ameliorate suffering - If anesthesia, euthanasia, or any kind of animal sacrifice is part of the study, include briefly which substances and/or methods were applied   **Field Research**  Include the following details if this study involves the collection of plant, animal, or other materials from a natural setting:   - Field permit number - Name of the institution or relevant body that granted permission |  |
| --- | --- |
|  |  |
| **Data Availability**  Authors are required to make all data underlying the findings described fully available, without restriction, and from the time of publication. PLOS allows rare exceptions to address legal and ethical concerns. See the [PLOS Data Policy](http://journals.plos.org/plosone/s/data-availability) and [FAQ](http://journals.plos.org/plosone/s/data-availability#loc-faqs-for-data-policy) for detailed information. | No - some restrictions will apply |

| A Data Availability Statement describing where the data can be found is required at submission. Your answers to this question constitute the Data Availability Statement and **will be published in the article**, if accepted. |  |
| --- | --- |
| **Important:** Stating ‘data available on request from the author’ is not sufficient. If your data are only available upon request, select ‘No’ for the first question and explain your exceptional situation in the text box. |  |
| Do the authors confirm that all data underlying the findings described in their manuscript are fully available without restriction? |  |
| **Describe where the data may be found in full sentences. If you are copying our sample text, replace any instances of XXX with the appropriate details.**   - If the data are **held or will be held in a public repository**, include URLs, accession numbers or DOIs. If this information will only be available after acceptance, indicate this by ticking the box below. For example: *All XXX files are available from the XXX database (accession number(s) XXX, XXX.*). - If the data are all contained **within the manuscript and/or Supporting Information files**, enter the following: *All relevant data are within the manuscript and its Supporting Information files.* - If neither of these applies but you are able to provide **details of access elsewhere**, with or without limitations, please do so. For example:   *Data cannot be shared publicly because of [XXX]. Data are available from the XXX Institutional Data Access / Ethics Committee (contact via XXX) for researchers who meet the criteria for access to confidential data.*  *The data underlying the results presented in the study are available from (include the name of the third party* | The data underlying the results presented in the study are available from corresponding author(xhl6363@sina.com) |

| *and contact information or URL).*   - This text is appropriate if the data are owned by a third party and authors do not have permission to share the data.   * typeset |  |
| --- | --- |
| Additional data availability information: |  |

Manuscript	[Click here to access/download;Manuscript;PLOS ONE.docx](https://www.editorialmanager.com/pone/download.aspx?id=25537501&guid=61708514-82e0-4fbd-9b13-4109211086fe&scheme=1)

# 1 The prevalence of poor sleep quality and associated risk factors among Chinese

2 **elderly adults in nursing homes: a cross-sectional study**
3 Zhao Hu1, ¶, Xidi Zhu1, ¶, Yunhan Yu^1^, Huilan Xu^1,*^
4 1 Department of Social Medicine and Health Management, Xiangya School of Public Health,
5 Central South University, Changsha, China
6 *Corresponding author
7 E-mail [address:xhl6363@sina.com](mailto:xhl6363@sina.com)
8 ¶ These authors contributed equally to this work

9

# 10 Abstract

11 **Background:** Sleep problems have become the most common complaints among elderly
12 adults. There are a few studies indentified prevalence of poor sleep quality and its associated
13 factors in nursing home setting.Therefore, our study aim to examine the prevalence of poor
14 sleep quality, its risk factors, and their interactions among Chinese elderly adults in nursing
15 homes.
16 **Methods:** A total of 817 elderly residents from 24 nursing homes were included in this
17 cross-sectional study. Sleep quality was assessed using the Pittsburgh Sleep Quality Index
18 (PSQI), and poor sleep quality was defined as PSQI >5. Multiple binary logistic regression
19 was used to estimate adjusted odds ratios (AORs) and 95% confidence intervals (CIs)
20 between risk factors and poor sleep quality. An additional interaction model was used to
21 analyse the interaction between risk factors.
22 **Results:** The prevalence of poor sleep quality was 67.3% (95% CI: 64.0, 70.5%) among
23 elderly adults in nursing homes. Multiple binary logistic regression results showed that
24 participants with the following characteristics had an increased risk of poor sleep quality
25 after adjustments for other confounders: being 70-79 years old (AOR: 1.81, 95% CI: 1.10,
26 2.97) or 80 years old and above (AOR: 2.64, 95% CI: 1.67, 4.17); having less than 7 years of
27 education (AOR: 1.59, 95% CI: 1.08, 2.33); having one to two kinds of chronic diseases (AOR:
28 2.26, 95% CI: 1.53, 3.32) or three or more kinds of chronic disease (AOR: 2.81, 95% CI: 1.65,

29	4.76); having depression (AOR: 3.13, 95% CI: 2.04, 4.81), anxiety (AOR: 3.42, 95% CI: 1.68,

30 6.97), and lower social support (AOR: 1.57, 95% CI: 1.11, 2.21). Additive interactions were
31 detected between age and anxiety (AOR: 8.34, 95% CI: 4.43, 15.69), between chronic disease
32 and anxiety (AOR: 8.61, 95% CI; 4.28, 17.31) and between social support and anxiety (AOR:

33	6.43, 95% CI: 3.22, 12.86).

34 **Conclusions**: The prevalence of poor sleep quality in nursing homes is relatively high.
35 Anxiety has additive interactions with age, chronic disease and social support for poor sleep
36 quality. These findings have significant implications for interventions that aim to improve
37 sleep quality among elderly residents in nursing homes.
38 **Keywords:** sleep quality; nursing home; interaction; risk factors

39

40

41

42

43

44

# 45 1.Introduction

46 Ageing is a severe problem in China as well as throughout the world. According to the
47 data of the Sixth National Population Census in 2010, the number of people in China aged 60
48 years and above was approximately 177 million, accounting for 13.26% of the total
49 population[1]. The U.N. Commission On Population Development has predicted that the
50 elderly population aged 60 and above in China will reach 243.8 million by 2020, accounting
51 for 17.1% of the total population [2]. Sleep problems have become the most common
52 complaints among elderly adults as they age. Approximately 74% of elderly men and 79% of
53 elderly women reported sleep complaints in Italy[3]. In addition, studies have reported that
54 the prevalence of sleep disorders was approximately 30-40% among older residents[4, 5].
55 However, as a complicated phenomenon, the sleep quality of individuals is difficult to define
56 and assess objectively. Using a standardized assessment tool for sleep quality named the
57 Pittsburgh Sleep Quality Index (PSQI), epidemiological studies have indicated that the
58 self-rated prevalence of poor sleep quality was 62.4% among older adults in Thailand[6],
59 16.6% in Mexican Americans aged 75 and older[7], 41.5% and 33.8% among elderly adults
60 living in urban and rural areas in China, respectively[8, 9]. However, several studies
61 demonstrated that most sleep problems may be exacerbated by institutional setting[10, 11].
62 For instance, a study conducted among 100 selected individuals over 65 years of age found
63 that institutionalized elderly adults presented worse overall sleep quality and higher levels
64 of daytime somnolence than non-institutionalized elderly adults[10]. Fetveit and colleagues
65 [11] found that the prevalence of sleep disturbance was approximately 70% among nursing
66 home residents. In summary, sleep problems are very common among elderly adults and
67 pose a major challenge to public health.
68 Evidence is emerging that poor sleep quality is positively associated with physical,
69 mental disorders and quality of life[12, 13]. A large cohort study found that poor sleep
70 quality was associated with a higher odds of hypertension in a Chinese rural population[13].
71 Another large cohort study indicated that short sleepers with poor sleep quality had an
72 increased risk of cardiovascular disease[14]. In addition, many studies[15-17] have indicated
73 that poor sleep quality is a powerful predictor of suicidal ideation and depressive symptoms
74 among elderly adults. However, the underlying mechanisms between poor sleep quality and
75 these harmful consequences are still unclear.
76 To alleviate the personal suffering and adverse effects introduced by poor sleep quality,
77 it is essential to understand its prevalence pattern and associated factors. Previous studies
78 have demonstrated many factors related to poor sleep quality among older adults, including
79 but not limited to the following four domains: (1) socio-demographic factors, such as age[9],
80 marital status[18] and education[19]; (2) lifestyle factors, such as physical activity [20] and
81 caffeine intake[21]; (3) emotional factors, such as stress[22] and depression[20]; and (4)
82 chronic conditions, such as arthritis[9] and pulmonary disease[23]. However, few studies
83 have examined the prevalence and risk factors of poor sleep quality in a nursing home
84 population. Moreover, no study has explored the interactions of risk factors for poor sleep
85 quality among elderly residents in nursing homes. Thus, the purposes of our study were as
86 follows: (1) to examine the prevalence of poor sleep quality among elderly adults in nursing
87 homes in China; (2) to analyse the risk factors for poor sleep quality; and (3) to explore the
88 interactions of risk factors for poor sleep quality.

# 89 2. Materials and methods

90 **2.1 Study population**
91 This was a cross-sectional study conducted in nursing homes in Hunan Province in China
92 from October to December 2018. To select a representative elderly sample living in nursing
93 homes of Hunan Province, a multistage sampling method was used. First, each city from
94 northern Hunan, southern Hunan and central Hunan was selected (i.e., based on
95 geographical region): Changsha city, Hengyang city and Yiyang city, respectively.
96 Subsequently, one county was randomly chosen from each selected city: Changsha County,
97 Hengyang County and Yuanjiang County. Second, two districts (Kaifu and Yuelu) from
98 Changsha city and two townships (Xingsha and Tiaoma) from Changsha County were
99 randomly selected. Similarly, Yanfeng and Shigu from Hengyang city and Xidu and Jingtou
100 from Hengyang County were randomly selected. Ziyang and Heshan from Yiyang city and
101 Qionghu and Caowei from Yuanjiang County were randomly chosen. Finally, two nursing
102 homes were randomly selected from each selected district and township, and a total of 24
103 nursing homes were ultimately selected.
104 Resident populations in selected nursing homes were included in our study if they met
105 the following inclusion criteria: (1) age 60 years and above; (2) duration of entrance into
106 nursing homes of more than one year; and (3) physical and mental ability to participate in
107 interviews. Participants were excluded if they (1) refused to participate in this study, (2) had
108 a severe hearing impairment or language barrier, and (3) had a history of severe cognitive
109 deficit. In summary, among a total of 2,055 older adults residents in the 24 nursing homes,
110 511 adults were excluded because they were younger than 60 years old or had stayed in a
111 nursing home less than one year. A total of 603 older adults had a severe hearing impairment,
112 language barrier or severe cognitive deficit, and 112 older adult residents who did not agree
113 to participate were also excluded from this study. Of the remaining 829 older adults, 12 were
114 excluded due to incomplete data. Ultimately, a total of 817 elderly adults were included in the
115 data analysis in this study.

# 116 2.2 Data measurement

117 All data were obtained through face-to-face interviews by trained staff who spent 30
118 minutes to 1 hour following a structured self-administrate questionnaire. We collected data
119 on socio-demographics (age, gender, education, marital status, monthly individual income,
120 number of descendants, length of stay in a nursing home and medical insurance status),
121 lifestyle behavioural factors (smoking, alcohol drinking and physical exercise), physical
122 status (activities of daily living (ADLs) and number of chronic disease) and social
123 psychological factors (social support, depression and anxiety). Marital status was
124 dichotomized into stable and unstable. Unstable marital status included divorce, the loss of a
125 partner and never having been married. Smoking was defined as an average of at least one
126 cigarette per day in the last year. Alcohol drinking was defined as drinking a glass of wine per
127 day in the last week. Regular physical exercise refers to engaging in at least 30 minutes of
128 physical exercise at least three times a week. ADL status was assessed using Lawton and
129 Brody’s ADL scale, which subsequently dichotomized participants into disabled and
130 normal[24]. Disabled ADL status was determined when participants had a total score of
131 greater than 14 points. Self-reported chronic diseases refer to chronic non-communicable
132 diseases, including hypertension, diabetes, coronary heart disease (CHD), chronic
133 obstructive pulmonary disease (COPD), hyperlipidaemia, stroke and others. Social support
134 was examined using the Social Support Rating Scale (SSRS), which was developed by Xiao
135 and is widely used in China. Then, participants were divided into two groups based on the
136 median of the global score (lower: score≤30 points; higher: score>30 points). Depression
137 was assessed using the Chinese version of the Geriatric Depression Scale-30 items (GDS-30)
138 [25], which indicated high validity and reliability among the Chinese elderly population[26].
139 The GDS-30 consists of 30 true/false questions, and the total score ranges from 0 to 30
140 points. Participants who scored 11 and above were considered to be depressive in this study.
141 Anxiety was assessed using the Chinese version of the Generalized Anxiety Disorder-7 scale
142 (GAD-7). The GAD-7 is a seven-item self-report instrument, and each item assesses one of the
143 typical symptoms of GAD over the last two weeks, with a total score that ranges from 0 to 21.
144 Participants who scored 10 and above were considered to be anxious in this study [27].

# 145 2.3 Assessment of sleep quality

146 Sleep quality among elderly residents in nursing homes was assessed using the Chinese
147 version of the Pittsburgh Sleep Quality Index (PSQI)[28], which has been proven to have
148 good validity and reliability among older adults[29]. The PSQI is a self-rated questionnaire
149 that assesses sleep quality in the past month. It contains nineteen items grouped into seven
150 components: subjective sleep quality, sleep latency, sleep duration, sleep efficiency, sleep
151 disturbances, use of sleeping medication, and daytime dysfunction. Each component score
152 ranges from 0 to 3, and the total score ranges from 0 to 21 points. Participants with a global
153 PSQI score greater than 5 were defined as having poor sleep quality in this study.

# 154 2.4 Statistical analysis

155 Categorical variables are presented as frequencies and percentages. Continuous
156 variables are presented as the mean±standard deviation (SD). The differences between
157 continuous variables were compared using Student’s t-test or one-way analysis of variance
158 (ANOVA). The differences between categorical variables were analysed using the Pearson
159 chi-square test or the Mantel-Haenszel chi-square test. Multiple binary logistic regression
160 analysis was used to examine risk factors associated with poor sleep quality. The adjusted
161 odds ratios (AORs) and 95% confidence intervals (CIs) were also estimated after controlling
162 for other confounders. Microsoft Excel, developed by Andersson and colleagues[30], was
163 used to detect and calculate the additive interaction of risk factors for poor sleep quality. The
164 relative excess risk due to interaction (RERI) and the attributable proportion due to
165 interaction (AP) being equal to 0 or the synergy index (S) being equal to 1 suggest no
166 additive interaction between the two factors. *P*<0.05 was considered to be statistically
167 significant. All statistical analyses were analysed using SAS 9.3 (SAS Institute Inc., Cary, NC,
168 USA).

# 169 3. Results

170 **3.1 Characteristics of the study sample**
171 In total, 817 elderly residents living in nursing homes were included in this study.
172 Among them, more than half of participants aged 80 years and above. A total of 54.0% of
173 subjects were female, and 46.0% were male. Approximately 40% of the participants had
174 completed less than 6 years of education and had stable marriages. Approximately one-third
175 reported monthly individual incomes of more than 3,000 RMB. A minority of participants
176 engaged in cigarette smoking and alcohol drinking and had no chronic diseases.
177 Approximately 36.0% of individuals had depressive symptoms, and 16.0% had anxiety
178 symptoms.
179 There were significant statistically significant differences between poor sleepers and
180 good sleepers in age, education, marital status, monthly individual income, length of stay,
181 number of chronic diseases, depression, anxiety, social support and ADL status (all *P*<0.05).
182 Poor sleepers were seemingly those who were older in age, had lower education, had
183 unstable marriages, had lower monthly individual incomes, had longer lengths of stay, had at
184 least one type of chronic disease, had depression, had anxiety, had lower social support and
185 had disabled ADLs. The results are presented in Table 1.
187 Table 1 Characteristics of elderly residents in nursing homes in this study

Characteristic

Total (n=817)

Poor sleeper (n=550)

Good sleeper (n=267)

*P*-value^†^

| Age (years) |  | | | |
| --- | --- | --- | --- | --- |
| 60~ | 135(16.5) | 73(13.3) | 62(23.2) | <0.001 |
| 70~ | 232(28.4) | 150(27.3) | 82(30.7) |  |
| 80~ | 450(55.1) | 327(59.4) | 123(46.1) |  |
| Gender |  |  |  |  |
| Male | 376(46.0) | 251(45.6) | 125(46.8) | 0.751 |
| Female | 441(54.0) | 299(54.4) | 142(53.2) |  |
| Education (years) |  |  |  |  |
| 0-6 | 364(44.6) | 263(47.8) | 101(37.8) | 0.004 |
| 7~ | 203(24.8) | 138(25.1) | 65(24.4) |  |
| 10~ | 250(30.6) | 149(27.1) | 101(37.8) |  |
| Marital status |  |  |  |  |
| Stable | 302(37.0) | 186(33.8) | 116(43.4) | 0.007 |
| Unstable | 515(63.0) | 364(66.2) | 151(56.6) |  |
| Medical insurance |  |  |  |  |
| Yes | 766(93.8) | 517(94.0) | 249(93.3) | 0.681 |
| No | 51(6.2) | 33(6.0) | 18(6.7) |  |
| Monthly individual income | | | | |
| ≤3,000 RMB | 568(69.5) | 404(73.5) | 164(61.4) | <0.001 |
| >3,000 RMB | 249(30.5) | 146(26.5) | 103(38.6) |  |
| Length of stay (years) |  |  |  |  |
| 1~ | 444(54.3) | 284(51.6) | 160(59.9) | 0.026 |
| 3~ | 373(45.7) | 266(48.4) | 107(40.1) |  |
| Have descendants |  |  |  |  |
| Yes | 746(91.3) | 496(90.2) | 250(93.6) | 0.100 |
| No | 71(8.7) | 54(9.8) | 17(6.4) |  |
| Smoking |  |  |  |  |
| Yes | 132(16.2) | 82(14.9) | 50(18.7) | 0.164 |
| No | 685(83.8) | 468(85.1) | 217(81.3) |  |
| Alcohol drinking |  |  |  |  |

Yes	96(11.8)	60(10.9)	36(13.5)	0.284

No	721(88.2)	490(89.1)	231(76.5)

Physical exercise

| Regular | 353(43.2) | 233(42.4) | 120(44.9) | 0.485 |
| --- | --- | --- | --- | --- |
| Irregular | 464(56.8) | 317(57.6) | 147(55.1) |  |
| Number of chronic diseases | | | | |
| 0 | 176(21.5) | 86(15.6) | 90(33.7) | <0.001 |
| 1~ | 471(57.7) | 328(59.7) | 143(53.6) |  |
| 3~ | 170(20.8) | 136(24.7) | 34(12.7) |  |
| ADL status |  |  |  |  |
| Normal | 268(32.8) | 152(27.6) | 116(43.4) | <0.001 |
| Disabled | 549(67.2) | 398(72.4) | 151(56.6) |  |
| Depression |  |  |  |  |
| Yes | 294(36.0) | 251(45.6) | 43(16.1) | <0.001 |
| No | 523(64.0) | 299(54.4) | 224(83.9) |  |
| Anxiety |  |  |  |  |
| Yes | 136(16.6) | 124(22.5) | 12(4.5) | <0.001 |
| No | 681(83.4) | 426(77.5) | 255(95.5) |  |
| Social support |  |  |  |  |
| Low | 435(53.2) | 337(61.3) | 98(36.7) | <0.001 |
| High | 382(46.8) | 213(38.7) | 169(63.3) |  |

188 Data are presented as n(%). ADLs, activities of daily living.
189 † P-value was determined by the Pearson chi-square test.

# 191 3.2 Prevalence of poor sleep quality

192 The mean global PSQI score was 8.5 points overall (95% CI: 8.2, 8.8), 8.3 points in males
193 (95% CI: 7.8, 8.8) and 8.7 points in females (95% CI: 8.2, 9.1). There was no statistically
194 significant difference in the PSQI score between males and females (*P*=0.293). The total PSQI
195 score increased with increasing age, from 7.4 points (95% CI: 6.6, 8.2) in subjects aged 60-69
196 years to 8.9 points (95% CI: 8.4, 9.3) in subjects aged ≥80 years (*P* for trend=0.010). A total
197 of 550 individuals had a total PSQI score greater than 5, and the overall prevalence of poor
198 sleep quality was 67.3% (95% CI: 64.0, 70.5%). The prevalence of poor sleep quality was

199	66.8% (95% CI: 61.7, 71.0%) in males and 67.8% (95% CI: 63.5, 72.1%) in females. The

200 prevalence rate was also increased by increasing age, ranging from 54.1% (95% CI: 45.9,
201 63.0%) in participants aged 60-69 years to 72.7% (95% CI: 68.7, 77.1%) in participants aged
202 ≥80 years (*P* for trend <0.001). The results are shown in Table 2.
204 Table 2 The PSQI global score and prevalence of poor sleep quality in nursing homes
205 stratified by age and gender

|  | N | PSQI score  Mean(95% CI) | P-value | Poor sleep quality^†^  Prevalence(95% CI) | P-value |
| --- | --- | --- | --- | --- | --- |
| Total Gender  Male | 817  376 | 8.5(8.2, 8.8)  8.3(7.8, 8.8) | 0.293 | 67.3(64.0, 70.5)  66.8(61.7, 71.0) | 0.751 |
| Female | 441 | 8.7(8.2, 9.1) |  | 67.8(63.5, 72.1) |  |

Age

| 60~ | 135 | 7.4(6.6, 8.2) | 0.010^*^ | 54.1(45.9, 63.0) | <0.001^*^ |
| --- | --- | --- | --- | --- | --- |
| 70~ | 232 | 8.4(7.8, 9.1) |  | 64.7(58.6, 70.5) |  |
| 80~ | 450 | 8.9(8.4, 9.3) |  | 72.7(68.7, 77.1) |  |

206 †Poor sleep quality was determined by PSQI score >5.
207 **P*-value for trend.

# 209 3.3 Components of sleep quality

210 Most of the participants went to bed at 9 PM and woke up at approximately 6 AM. The
211 average bedtime of the participants was 9.1±3.5 hours, and the average sleep duration was
212 6.8±3.9 hours. Most subjects could not fall asleep within 30 minutes. The average sleep
213 efficiency of the participants was 80.0%. Approximately 85% of subjects never used sleep
214 medication. The mean scores for subjective sleep quality, sleep disturbance and daytime
215 dysfunction were 1.3±0.9, 1.2±0.6 and 1.4 ±1.1, respectively.
216 No statistically significant differences were observed between males and females on all
217 components of sleep quality. More older residents than younger people have used sleep
218 medication. Moreover, older adults have more daytime dysfunction than younger individuals
219 do. No significant differences were obtained in sleep latency, sleep efficiency or sleep
220 disturbance between age groups. The results are shown in Table 3.
221 Table 3 Gender- and age-specific scores of sleep quality components measured by the Chinese version of the Pittsburgh Sleep Quality Index

Sleep quality components

Total

Gender	Age group (years)

(n=817)

|  | | | (n=376) | (n=441) |  | (n=135) | (n=232) | (n=450) |  |
| --- | --- | --- | --- | --- | --- | --- | --- | --- | --- |
| Subjective sleep quality^†^, mean±SD |  | 1.3±0.9 | 1.3±0.9 | 1.3±0.9 | 0.674 | 1.2±0.9 | 1.3±0.9 | 1.3±0.9 | 0.482 |
| Sleep latency, n(%) | ≤15 min | 236(28.9) | 116(30.9) | 120(27.2) | 0.200 | 51(37.8) | 63(27.2) | 122(27.1) | 0.161 |
|  | 16~min | 252(30.8) | 121(32.2) | 131(29.7) |  | 34(25.2) | 75(32.3) | 143(31.8) |  |
|  | 31~min | 329(40.3) | 139(36.9) | 190(43.1) |  | 50(37.0) | 94(40.5) | 185(41.1) |  |
| Sleep duration, mean±SD |  | 6.8±3.9 | 6.8±3.1 | 6.8±4.4 | 0.908 | 6.9±2.1 | 6.7±2.7 | 6.9±4.7 | 0.759 |
| Sleep duration, n(%) | <6 h | 262(32.1) | 121(32.2) | 141(32.0) | 0.253 | 38(28.1) | 80(34.5) | 144(32.0) | 0.008 |
|  | 6-7 h | 278(34.0) | 118(31.4) | 160(36.3) |  | 38(28.1) | 67(28.9) | 173(38.4) |  |
|  | >7 h | 277(33.9) | 137(36.4) | 140(31.7) |  | 59(43.8) | 85(36.6) | 133(29.6) |  |
| Habitual sleep efficiency^*^, mean±SD |  | 0.8±0.4 | 0.8±0.3 | 0.8±0.4 | 0.593 | 0.8±0.2 | 0.7±0.2 | 0.8±0.5 | 0.558 |
| Habitual sleep efficiency, n(%) | <65% | 270(33.0) | 125(33.2) | 145(32.8) | 0.803 | 41(30.4) | 80(34.5) | 149(33.1) | 0.075 |
|  | 65-74% | 103(12.6) | 48(12.8) | 55(12.5) |  | 14(10.4) | 28(12.1) | 61(13.6) |  |
|  | 75-84% | 135(16.5) | 57(15.2) | 78(17.7) |  | 15(11.1) | 34(14.7) | 86(19.1) |  |
|  | >85% | 309(37.8) | 146(38.8) | 163(37.0) |  | 65(48.1) | 90(38.8) | 154 (34.2) |  |
| Sleep disturbances^‡^, mean±SD |  | 1.2±0.6 | 1.2±0.6 | 1.2±0.6 | 0.914 | 1.2±0.6 | 1.3±0.6 | 1.3±0.5 | 0.148 |
| Any sleep disturbance, n(%) | Yes | 248(30.4) | 112(29.8) | 136(30.8) | 0.745 | 38(28.1) | 72(31.0) | 138(30.7) | 0.826 |
|  | No | 569(69.6) | 264(60.2) | 305(69.2) |  | 97(71.9) | 160(69.0) | 312(69.3) |  |
| Use of sleep medication, n(%) | Yes | 115(14.1) | 44(11.7) | 71(16.1) | 0.072 | 9(6.7) | 23(9.9) | 83(18.4) | <0.001 |
|  | No | 702(85.9) | 332(88.3) | 370(83.9) |  | 126(93.3) | 209(90.1) | 367(81.6) |  |
| Daytime dysfunction^**^, mean±SD |  | 1.4±1.1 | 1.4±1.1 | 1.5±1.0 | 0.620 | 1.2 ±1.0 | 1.5±1.1 | 1.5±1.0 | 0.037 |

Male

Female

P-value	60-69

70-79

≥80

P-value

222 SD,	*Derived from the formula: hours of sleep/(get-up
223 time−usual bedtime)×100%; score range from 0 to 1; higher scores indicate higher sleep efficiency; ‡Derived from PSQI items 5b-5j; score range from 0 to 3; higher
224**Derived from PSQI item 8 and 9; score range from 0 to 3; higher scores indicate more daytime dysfunction.

# 225 3.4 Risk factors for poor sleep quality

226 Multivariate binary logistic regression analysis results suggest that age between 70 to 79

227	years (AOR: 1.81, 95% CI: 1.10, 2.97) or 80 years and above (AOR: 2.64, 95% CI: 1.67, 4.17)

228 increased the risk of poor sleep quality compared to age between 60 and 69 years after
229 adjustments were made for other confounding factors. Moreover, similar results also
230 indicated that participants with 0-6 years of education (AOR: 1.59, 95% CI: 1.08, 2.33), one
231 to two kinds of chronic diseases (AOR: 2.26, 95% CI: 1.53, 3.32) or three or more kinds of

232	chronic diseases (AOR: 2.81, 95%: 1.65, 4.76), depression (AOR: 3.13, 95% CI: 2.04, 4.81) or

233 anxiety (AOR: 3.42, 95% CI: 1.68, 6.97) and lower social support (AOR: 1.57, 95% CI: 1.11,
234 2.21) were more likely to report poor sleep quality after adjustments were made for other
235 variables. The results are shown in Table 4.
237 Table 4 Risk factors associated with poor sleep quality among elderly adults in nursing
238 homes

| Variable | B | SE | Wald | AOR(95% CI)^†^ | P-value |
| --- | --- | --- | --- | --- | --- |
| Age (years) |  |  |  |  |  |
| 60~ |  |  |  | 1.00 |  |
| 70~ | 0.59 | 0.25 | 5.51 | 1.81(1.10, 2.97) | 0.019 |
| 80~ | 0.97 | 0.23 | 17.32 | 2.64(1.67, 4.17) | <0.001 |
| Education (years) |  |  |  |  |  |
| 10~ |  |  |  | 1.00 |  |
| 7~ | 0.34 | 0.22 | 2.28 | 1.40(0.90, 2.16) | 0.132 |
| 0-6 | 0.46 | 0.20 | 5.49 | 1.59(1.08, 2.33) | 0.019 |
| Number of chronic diseases | | | | | |
| 0~ |  |  |  | 1.00 |  |
| 1~ | 0.82 | 0.20 | 17.02 | 2.26(1.53, 3.32) | <0.001 |
| 3~ | 1.03 | 0.27 | 14.61 | 2.81(1.65, 4.76) | <0.001 |
| Depression |  |  |  |  |  |
| No |  |  |  | 1.00 |  |
| Yes | 1.14 | 0.22 | 27.05 | 3.13(2.04, 4.81) | <0.001 |
| Anxiety |  |  |  |  |  |
| No |  |  |  | 1.00 |  |
| Yes | 1.23 | 0.36 | 11.53 | 3.42(1.68, 6.97) | 0.001 |
| Social support |  |  |  |  |  |
| High |  |  |  | 1.00 |  |
| Low | 0.45 | 0.18 | 6.51 | 1.57(1.11, 2.21) | 0.011 |

239 SE, standard error; AOR, adjusted odds ratio.
240 †Adjusted for gender, marital status, smoking, alcohol drinking, physical exercise, descendants,
241 medical insurance, monthly individual income, length of stay and ADL status, as well as the
242 variables in the table.

# 244 3.5 Interactions of risk factors for poor sleep quality

245 In this study, Andersson’s Excel was used to detect the interactions and calculate the
246 effect size between risk factors for poor sleep quality. The adjusted factors included all
247 variables (except for two), the interactions among which we would examine. Additional
248 interactions were detected between the following variables: older age and anxiety, with an

249 RERI of 2.83 (95% CI: 0.13, 5.52), an AP of 0.34 (95% CI: 0.13, 0.55) and an S of 1.63 (95% CI:

250 1.11, 2.39); chronic disease and anxiety, with an RERI of 3.32 (95% CI: 0.31, 6.33), an AP of 251	0.39 (95% CI: 0.29, 0.48) and an S of 1.77 (95% CI: 1.54, 2.04); and social support and	252 anxiety, with an RERI of 2.96 (95% CI: 1.02, 4.90), an AP of 0.46 (95% CI: 0.38, 0.54) and an S

253 of 2.20 (95% CI: 1.65, 2.93). No statistically significant interactions were found between
254 other factors after adjusting for confounders. The results are shown in Table 5.
256 Table 5 Interactions of risk factors for poor sleep quality among elderly adults in nursing
257 homes

| Factor 1 | Factor 2 | AOR^†^ | RERI | AP | S |
| --- | --- | --- | --- | --- | --- |
| Age (years) | Anxiety |  | 2.83(0.13, 5.52) | 0.34(0.13, 0.55) | 1.63(1.11, 2.39) |
| 60-69 | No | 1.00 |  |  |  |
| ≥70 | No | 2.09(1.01, 4.32) |  |  |  |
| 60-69 | Yes | 4.43(2.13, 9.20) |  |  |  |
| ≥70  Chronic disease | Yes  Anxiety | 8.34(4.43, 15.69) | 3.32(0.31, 6.33) | 0.39(0.29, 0.48) | 1.77(1.54, 2.04) |
| No | No | 1.00 |  |  |  |
| Yes | No | 2.51(1.92, 3.29) |  |  |  |
| No | Yes | 3.79(1.82, 7.86) |  |  |  |
| Yes | Yes | 8.61(4.28, 17.31) |  |  |  |
| Social support | Anxiety |  | 2.96(1.02, 4.90) | 0.46(0.38, 0.54) | 2.20(1.65, 2.93) |
| High | No | 1.00 |  |  |  |
| Low | No | 1.61(1.10, 2.35) |  |  |  |
| High | Yes | 2.87(1.38, 5.95) |  |  |  |
| Low | Yes | 6.43(3.22, 12.86) |  |  |  |

258 AOR, adjusted odds ratio; RERI, the relative excess risk due to interaction; AP, the attributable
259 proportion due to interaction; S, the synergy index.
260 †Adjusted for gender, education, marital status, smoking, alcohol drinking, physical exercise,
261 descendants, medical insurance, monthly individual income, length of stay, depression and ADL
262 status, as well as the variables in the table.

# 264 4. Discussion

265 In this study, we examined the prevalence of poor sleep quality and its risk factors among
266 elderly adults in nursing homes in China. Additionally, we explored the interactions of risk
267 factors for poor sleep quality. We found that poor sleep quality affected nearly two-thirds of
268 elderly residents in nursing homes and that older age, lower education, chronic diseases,
269 depression, anxiety and lower social support were risk factors for poor sleep quality.
270 Moreover, additive interactions were detected between age and anxiety and between
271 education and anxiety.
272 This large population-based study used a standardized and valid PSQI tool to measure
273 the characteristics of sleep quality among elderly adults in nursing homes in China. Based on
274 this approach, we found that the prevalence of poor sleep quality was 67.3% among elderly
275 residents in nursing homes in China. This finding is comparable to those of other previous
276 studies conducted in elderly adults living in nursing homes in Turkey[31, 32]. However, with
277 a similar population, the same institutional setting and measurement tool, Stefan et al [33]
278 found that 54.5% of individuals were poor sleepers among 894 elderly adults in Zagreb
279 nursing homes. Tsai et al [34] found that 46.4% of participants reported poor sleep quality
280 among 196 elderly nursing home residents in Taiwan. A reason for the variance in these
281 estimates may be explained by the different inclusion criteria for the study sample, as well as
282 the differences in facilities and medical care among different institutions. As mentioned in
283 the introduction, some sleep problems may be exacerbated by institutional setting. Our study
284 also indicates that the prevalence of poor sleep quality in nursing homes was higher than in
285 community homes. For instance, only 33.8% of community-dwelling elderly adults in Yanggu
286 County[8] and 27.7% in Deqing County[35] in China reported poor sleep quality. On the one
287 hand, the high self-rated prevalence may result from residents in nursing homes being older
288 (55.1% of participants were aged 80 years and above in this study) and having more chronic
289 conditions (78.5% of participants have at least one kind of chronic disease) than elderly
290 adults in the community[36]. On the other hand, an uncomfortable environment and care
291 routines in the institution may not promote sleep[37]. Among the seven PSQI components,
292 lower sleep efficiency (62.2%) was the most common, followed by longer sleep latency
293 (40.3%) and sleep disturbances (30.4%). These results were consistent with those of
294 previous studies [31, 32]. As shown in our study, elderly adults suffered from several
295 negative emotions (e.g., depression and anxiety) that may lead to longer sleep latency and
296 lower sleep efficiency[38]. The main cause of sleep disturbances in nursing home settings
297 may be nocturia in elderly adults, as well as noise and an uncomfortable environment[39].
298 However, the use of sleep medication was not prevalent in this population, and only 14.1% of
299 individuals reported that they had taken sleep medication in the previous month. These
300 results indicated that sleep problems in residents may not attract enough attention by
301 administrative and healthcare staff in nursing homes. Otherwise, people had no awareness
302 and believed that they were obtaining healthcare services for sleep problems in such a
303 setting.
304 Consistent with previous epidemiological studies in the community, our study found
305 that the prevalence of poor sleep quality was higher in older participants [9, 40]. However,
306 the findings of studies on the association between sleep quality and gender were conflicting.
307 Many studies indicate that females are more prone than males to experience poor sleep
308 quality because a higher proportion of females have lower socioeconomic status and are
309 more susceptible to anxiety and depression[40, 41]. In contrast, some studies indicate that
310 males are more likely to report sleep problems[42, 43]. In our study, no significant difference
311 in prevalence was observed between males and females, which is consistent with the
312 findings of a study conducted among elderly attendees of a primary care centre in Malaysia
313 [44] and the findings of another study conducted in nursing homes in Taiwan[34]. The
314 differences in these findings may be related to the differences in the study sample and
315 research location. For other socio-demographic factors, a shorter education duration was
316 positively associated with poor sleep quality after adjustments were made for other
317 confounders. This finding is consistent with those of other previous studies in both
318 institutionalized and non-institutionalized elderly individuals. One possible reason is that
319 less educated elderly adults may have limited ability to obtain and utilize information to
320 manage some sleep problems, thus contributing to poor sleep quality. Specific chronic
321 conditions, such as hypertension[45], heart disease[44], arthritis[9] and COPD[8], were
322 found to be associated with poor sleep quality. In our current study, one interesting finding is
323 that the risk of poor sleep quality was increased with an increasing number of chronic
324 diseases. This result is in accordance with those of other previous studies. For example, a
325 cross-sectional study performed among 16,680 residents aged 65 years in eight low- and
326 middle-income countries demonstrated that poor health and a high number of comorbidities
327 were associated with more sleep complaints[46]. Another population-based cross-sectional
328 study among 5,107 adults in Japan suggested that the PSQI score increased linearly as the
329 number of comorbid conditions increased [47]. This phenomenon provides new insights into
330 this association, which may be caused by some type of cluster effect between multiple
331 chronic conditions and requires further investigation.
332 Consistent with the findings of previous studies, depression and anxiety independently
333 increased the risk of poor sleep quality[48-50]. For example, Cho HJ et al[51] found that a
334 greater level of depressive symptoms had increased odds of sleep latency ≥1 hour in 3,051
335 participants aged 67 and older in the US. Another study conducted among 2,040 elderly
336 Koreans suggested that poor subjective sleep quality, longer sleep latency and the frequent
337 use of sleeping medication were independently associated with depression[52]. Potvin and
338 colleague [53] suggest that nearly all PSQI subscales were significantly associated with
339 anxiety, especially those for daytime sleepiness and sleep disturbances. Press et al [54]
340 reported that depressive symptoms were associated with decreased sleep satisfaction, while
341 anxiety symptoms were associated with difficulty falling asleep, waking up during the night
342 and morning weakness. In addition, we also discovered that anxiety not only is an
343 independent risk factor for poor sleep quality but also has additive interactions with older
344 age and chronic disease. In other words, anxious participants who are also older in age or
345 who have a chronic disease would have a higher prevalence of poor sleep quality. Many
346 studies have reported that anxiety symptoms are more commonly seen in older people[55,
347 56]. Additionally, studies have indicated that chronic disease and anxiety symptoms occur
348 contemporaneously, which may reinforce each other and result in a much stronger
349 association[57, 58]. These results may partly explain these interactions between three risk
350 factors and provide new insights into the prevention and treatment of sleep problems.
351 We assessed social support using the SSRS, which is a self-rated questionnaire
352 developed by Xiao and widely used in epidemiological studies in China. This perceived social
353 support scale evaluates social relationships with friends, neighbours, colleagues and family
354 members as well as the availability of functional support. Many studies consider social
355 support to be an important factor in the maintenance of health status. In our study, we found
356 that a lower level of social support increased the risk of poor sleep quality in nursing home
357 residents. This result was consistent with the findings of previous studies[59-61]. For
358 example, Kishimoto et al [60] found that individuals with weak social support from spouses
359 or family members had significantly increased adjusted odds ratios for the risk of sleep
360 disturbances. Another study[61] conducted among 998 African-American elderly adults
361 indicated that those who reported lower levels of emotional social support have more
362 difficulties in initiating and maintaining sleep. First, social support may influence sleep
363 quality by enabling a feeling of belonging and protection from social isolation and negative
364 emotions (e.g., loneliness, depression and anxiety), thus decreasing the incident risk of sleep
365 disorders [62-64]. Similar to our study results, people with anxiety had an increased risk of
366 poor sleep quality, especially those with lower social support. Additionally, a stronger social
367 network may contribute to individuals having multiple sources of health information, thus
368 increasing the probability of obtaining information that may help maintain and promote
369 healthy sleep behaviour [65, 66].
370 To the best of our knowledge, this is the first study to explore the risk factors and their
371 interactions for poor sleep quality among elderly adults in nursing homes in China. With a
372 large population-based sample and a higher response rate, these findings of our study may
373 provide valuable information for sleep among aging populations in nursing homes. From a
374 clinical point of view, concern about mental health in residents and intervention and
375 treatment for anxiety symptoms may be helpful in the improvement of sleep quality in
376 nursing homes. Additionally, the amelioration of the living environment and the provision of
377 more social support were also important. However, there are also several limitations that
378 need to be addressed. First, the causation between some risk factors and poor sleep quality
379 cannot be inferred due to the cross-sectional study design. For example, some studies also
380 declared that poor sleep quality was positively associated with depression, anxiety and
381 chronic diseases[67, 68]. Second, all data were obtained through self-rating and may
382 introduce recall bias. Third, the limited sensitivity and specificity of the Chinese version of
383 PSQI might lead to misclassifications for poor sleepers. Finally, our study only provided
384 evidence on these associations among elderly adults in nursing homes. Therefore, these
385 limitations	need	to	be	fully	considered	when	generalizing	these	results	to
386 community-dwelling elderly adults.

# 387 5. Conclusions

388 The prevalence of poor sleep quality in nursing homes is relatively high. The risk factors
389 for poor sleep quality were older age, lower education, chronic diseases, depression, anxiety
390 and lower social support. Anxiety has additive interactions with age, chronic disease and
391 social support for poor sleep quality. These findings have significant implications for
392 interventions that aim to improve sleep quality among elderly residents in nursing homes.

# 393 Acknowledgements

394 We thank all elderly residents in nursing homes for their cooperation. This study was
395 approved by the Ethical Committee of Xiangya School of Public Health in Central South
396 University (No.XYGW-2018-49).Written informed consent was obtained from all participants
397 of this study.

# 398 Data Availability Statement

399 The datasets used and analysed during the current study are available from the
400 corresponding author on reasonable request

# 401 Consent for publication

402 Not applicable.

# 403 Competing interests

404 The authors declare that they have no competing interests.

# 405 References

406 1.	National Bureau of Statistics of China. The Sixth National Population Census Data Gazette
407 (No. 1). National Census Bureau. Beijing, China: 2011.
408 2.	National committee on aging.Report on prediction of population aging trend in China.
409 <http://www.cncaprc.gov.cn/contents/16/11224.html>.; 2007-12-27.
410 3.	Maggi S, Langlois JA, Minicuci N, Grigoletto F, Pavan M, Foley DJ, et al. Sleep complaints in
411 community-dwelling older persons: prevalence, associated factors, and reported causes. J Am

412	Geriatr Soc. 1998;46(2):161-8. Epub 1998/02/25. PubMed PMID: 9475443.

413	4.	Roepke SK, Ancoli-Israel S. Sleep disorders in the elderly. The Indian journal of medical

414	research. 2010;131:302-10. Epub 2010/03/24. PubMed PMID: 20308755.

415	5.	Roberts RE, Shema SJ, Kaplan GA, Strawbridge WJ. Sleep complaints and depression in an

416	aging cohort: A prospective perspective. The American journal of psychiatry. 2000;157(1):81-8.

417	Epub 2000/01/05. doi: 10.1176/ajp.157.1.81. PubMed PMID: 10618017.

418 6.	Aunjitsakul W, Pitanupong J, Werachattawan N. Sleep Quality among Elderly People in
419 Songkhla Province, Thailand: A Two-Stage Cluster Sampling Study. Journal of the Medical
420 Association of Thailand. 2018;101(1):137-44. PubMed PMID: BCI:BCI201800464697.
421 7.	Pedraza S, Al Snih S, Ottenbacher KJ, Markides KS, Raji MA. Sleep quality and sleep problems
422 in Mexican Americans aged 75 and older. Aging Clin Exp Res. 2012;24(4):391-7. Epub 423 2011/11/22. doi: 10.3275/8106. PubMed	PMID: 22102513; PubMed	Central	PMCID: 424 PMCPMC3521094.
425 8.	Wang P, Song L, Wang K, Han X, Cong L, Wang Y, et al. Prevalence and associated factors of
426 poor sleep quality among Chinese older adults living in a rural area: a population-based study.
427 Aging clinical and experimental research. 2019. doi: 10.1007/s40520-019-01171-0. PubMed
428 PMID: MEDLINE:30919262.
429 9.	Luo J, Zhu G, Zhao Q, Guo Q, Meng H, Hong Z, et al. Prevalence and Risk Factors of Poor Sleep
430 Quality among Chinese Elderly in an Urban Community: Results from the Shanghai Aging Study.

431	Plos	One.	2013;8(11).	doi:	10.1371/journal.pone.0081261.	PubMed	PMID:

432	WOS:000327543500078.

433	10. Martins	da	Silva	R, Afonso P, Fonseca	M, Teodoro	T. Comparing sleep quality in

434	institutionalized and non-institutionalized elderly individuals. Aging & mental health. 2019:1-7.

435	Epub 2019/05/18. doi: 10.1080/13607863.2019.1619168. PubMed PMID: 31099258.

436	11. Fetveit A, Bjorvatn B. Sleep disturbances among nursing home residents. International

437	journal of geriatric psychiatry. 2002;17(7):604-9. doi: 10.1002/gps.639. PubMed PMID:

438	WOS:000176887000002.

439	12. Uchmanowicz I, Markiewicz K, Uchmanowicz B, Koltuniuk A, Rosinczuk J. The relationship

440	between sleep disturbances and quality of life in elderly patients with hypertension. Clinical

441	interventions in aging. 2019;14:155-65. Epub 2019/01/31. doi: 10.2147/cia.s188499. PubMed

442	PMID: 30697040; PubMed Central PMCID: PMCPMC6339653.

443	13. Zhang H, Li Y, Zhao X, Mao Z, Abdulai T, Liu X, et al. The association between PSQI score and

444	hypertension in a Chinese rural population: the Henan Rural Cohort Study. Sleep medicine.

445	2019;58:27-34. Epub 2019/05/07. doi: 10.1016/j.sleep.2019.03.001. PubMed PMID: 31059971.

446	14. Hoevenaar-Blom MP, Spijkerman AM, Kromhout D, van den Berg JF, Verschuren WM. Sleep

447	duration and sleep quality in relation to 12-year cardiovascular disease incidence: the MORGEN

448	study. Sleep. 2011;34(11):1487-92. Epub 2011/11/02. doi: 10.5665/sleep.1382. PubMed PMID:

449	22043119; PubMed Central PMCID: PMCPMC3198203.

450	15. Gelaye B, Okeiga J, Ayantoye I, Berhane HY, Berhane Y, Williams MA. Association of suicidal

451	ideation with poor sleep quality among Ethiopian adults. Sleep & breathing = Schlaf & Atmung.

452	2016;20(4):1319-26. Epub 2016/10/25. doi: 10.1007/s11325-016-1418-9. PubMed PMID:

453	27771845; PubMed Central PMCID: PMCPMC5159269.

454	16. Livingston G, Blizard B, Mann A. Does sleep disturbance predict depression in elderly people?

455	A study in inner London. The British journal of general practice : the journal of the Royal College

456	of General Practitioners. 1993;43(376):445-8. Epub 1993/11/01. PubMed PMID: 8292414;

457	PubMed Central PMCID: PMCPMC1372481.

458	17. Sukegawa T, Itoga M, Seno H, Miura S, Inagaki T, Saito W, et al. Sleep disturbances and

459	depression in the	elderly	in	Japan. Psychiatry	Clin Neurosci. 2003;57(3):265-70. doi:

460	10.1046/j.1440-1819.2003.01115.x. PubMed PMID: WOS:000182651600004.

461	18. Stranges S, Tigbe W, Gomez-Olive FX, Thorogood M, Kandala NB. Sleep problems: an

462	emerging global epidemic? Findings from the INDEPTH WHO-SAGE study among more than

463	40,000 older adults from 8 countries across Africa and Asia. Sleep. 2012;35(8):1173-81. Epub

464	2012/08/02. doi: 10.5665/sleep.2012. PubMed PMID: 22851813; PubMed Central PMCID:

465	PMCPMC3397790.

466	19. Thichumpa W, Howteerakul N, Suwannapong N, Tantrakul V. Sleep quality and associated

467	factors among the elderly living in rural Chiang Rai, northern Thailand. Epidemiol Health.

468	2018;40:e2018018. Epub 2018/05/29. doi: 10.4178/epih.e2018018. PubMed PMID: 29807410;

469	PubMed Central PMCID: PMCPMC6060346.

470	20. Sagayadevan V, Abdin E, Binte Shafie S, Jeyagurunathan A, Sambasivam R, Zhang Y, et al.

471	Prevalence and correlates of sleep problems among elderly Singaporeans. Psychogeriatrics.

472	2017;17(1):43-51. doi: 10.1111/psyg.12190. PubMed PMID: WOS:000393967000006.

473	21. de Pooter-Stijnman LMM, Vrijkotte S, Smalbrugge M. Effect of caffeine on sleep and

474	behaviour	in	nursing	home	residents	with	dementia. European	geriatric medicine.

475	2018;9(6):829-35. Epub	2018/12/24. doi: 10.1007/s41999-018-0115-6. PubMed	PMID:

476	30574215; PubMed Central PMCID: PMCPMC6267648.

477	22. Quan SA, Li YC, Li WJ, Li Y, Jeong JY, Kim DH. Gender Differences in Sleep Disturbance among

478	Elderly Koreans: Hallym Aging Study. Journal of Korean medical science. 2016;31(11):1689-95.

479	Epub 2016/10/07. doi: 10.3346/jkms.2016.31.11.1689. PubMed PMID: 27709844; PubMed

480	Central PMCID: PMCPMC5056198.

481	23. Su TP, Huang SR, Chou P. Prevalence and risk factors of insomnia in community-dwelling

482	Chinese elderly: a Taiwanese urban area survey. The Australian and New Zealand journal of

483	psychiatry. 2004;38(9):706-13. Epub 2004/08/25. doi: 10.1080/j.1440-1614.2004.01444.x.

484	PubMed PMID: 15324335.

485	24. Lawton MP, Brody EM. Assessment of older people: self-maintaining and instrumental

486	activities	of	daily	living.	The	Gerontologist.	1969;9(3):179-86.	doi:

487	10.1093/geront/9.3_Part_1.179. PubMed PMID: MEDLINE:5349366.

488	25. Smalbrugge M, Jongenelis L, Pot AM, Beckman ATF, Eefsting JA. Screening for depression and

489	assessing change in severity of depression. Is the Geriatric Depression Scale (30-, 15- and 8-item

490	versions) useful for both purposes in nursing home patients? Aging & mental health.

491	2008;12(2):244-8. doi: 10.1080/13607860801987238. PubMed PMID: WOS:000255342900011.

492 26. Lu J, Wang Y, Wang X, Song R, Yi X. Reliability and Validity of the Chinese Version of Geriatric
493 Depression Scale Among Chinese Urban Community-dwelling Elderly Population. Chinese Journal
494 of Clinical Psychology. 2013;21(1):39-41.
495 27. Spitzer RL, Kroenke K, Williams JBW, Loewe B. A brief measure for assessing generalized
496 anxiety	disorder - The GAD-7. Archives of Internal Medicine. 2006;166(10):1092-7. doi:

497	10.1001/archinte.166.10.1092. PubMed PMID: WOS:000237720500008.

498	28. Buysse DJ, Reynolds CF, 3rd, Monk TH, Berman SR, Kupfer DJ. The Pittsburgh Sleep Quality

499	Index: a	new	instrument	for	psychiatric	practice	and	research. Psychiatry	research.

500	1989;28(2):193-213. doi: 10.1016/0165-1781(89)90047-4. PubMed PMID: MEDLINE:2748771.

501 29. Jian-qing Q, Mi Z, Pei-yuan Q, Na Z, Yang W. Reliability and Validity of the Pittsburgh Sleep
502 Quality Index in older adults in rural area. Modern Preventive Medicine. 2016;43(10):1835-8.In
503 Chinese.
504 30. Andersson T, Alfredsson L, Kallberg H, Zdravkovic S, Ahlbom A. Calculating measures of
505 biological interaction. European journal of epidemiology. 2005;20(7):575-9. Epub 2005/08/27.
506 PubMed PMID: 16119429.
507 31. Orhan FO, Tuncel D, Tas F, Demirci N, Ozer A, Karaaslan MF. Relationship between sleep
508 quality and depression among elderly nursing home residents in Turkey. Sleep and Breathing. 509	2012;16(4):1059-67.	doi:	10.1007/s11325-011-0601-2.	PubMed	PMID: 510 WOS:000311301700020.

511	32. Eser I, Khorshid L, Cinar S. Sleep quality of older adults in nursing homes in Turkey:

512	enhancing the quality of sleep improves quality of life. Journal of gerontological nursing.

513 2007;33(10):42-9. Epub 2007/10/25. PubMed PMID: 17955737.

514 33. Stefan L, Vrgoc G, Rupcic T, Sporis G, Sekulic D. Sleep Duration and Sleep Quality Are
515 Associated with Physical Activity in Elderly People Living in Nursing Homes. International journal
516 of	environmental	research	and	public	health. 2018;15(11). Epub	2018/11/15. doi:
517 10.3390/ijerph15112512. PubMed PMID: 30423981; PubMed Central PMCID: PMCPMC6266288.
518 34. Tsai YF, Wong TKS, Ku YC. Self-care management of sleep disturbances and risk factors for
519 poor sleep among older residents of Taiwanese nursing homes. Journal of clinical nursing. 520	2008;17(9):1219-26. doi: 10.1111/j.1365-2702.2007.02020.x. PubMed	PMID: 521 WOS:000254990600011.

522 35. Dong X, Wang Y, Chen Y, Wang X, Zhu J, Wang N, et al. Poor sleep quality and influencing

523	factors among rural adults in Deqing, China. Sleep & breathing = Schlaf & Atmung. 524 2018;22(4):1213-20. Epub 2018/06/25. doi: 10.1007/s11325-018-1685-8. PubMed PMID: 525 29936592.

526	36. Gnadinger M, Herzig L, Ceschi A, Conen D, Staehelin A, Zoller M, et al. Chronic conditions and

527 multimorbidity in a primary care population: a study in the Swiss Sentinel Surveillance Network

528 (Sentinella). International journal of public health. 2018;63(9):1017-26. Epub 2018/05/23. doi: 529	10.1007/s00038-018-1114-6. PubMed	PMID: 29786762; PubMed	Central	PMCID: 530 PMCPMC6245242.

531 37. Gentili A, Weiner DK, Kuchibhatil M, Edinger JD. Factors that disturb sleep in nursing home 532 residents. Aging (Milan, Italy). 1997;9(3):207-13. doi: 10.1007/bf03340151. PubMed PMID: 533 MEDLINE:9258380.

534	38. Gentili A, Weiner DK, Kuchibhatil M, Edinger JD. Factors that disturb sleep in nursing home

535	residents. Aging (Milano). 1997;9(3):207-13. Epub 1997/06/01. PubMed PMID: 9258380.

536	39. Ersser S, Wiles A, Taylor H, Wade S, Walsh R, Bentley T. The sleep of older people in hospital

537	and nursing homes. Journal of clinical nursing. 1999;8(4):360-8. Epub 2000/01/07. PubMed

538	PMID: 10624252.

539	40. Wang Y, Li Y, Liu X, Liu R, Mao Z, Tu R, et al. Gender-specific prevalence of poor sleep quality

540	and related factors in a Chinese rural population: the Henan Rural Cohort Study. Sleep medicine.

541	2019;54:134-41. Epub 2018/12/17. doi: 10.1016/j.sleep.2018.10.031. PubMed PMID: 30554057.

542	41. Kara B, Tenekeci E. Sleep Quality and Associated Factors in Older Turkish Adults With

543	Hypertension:	A	Pilot	Study.	J	Transcult	Nurs.	2017;28(3):296-305.	doi:

544	10.1177/1043659615623330. PubMed PMID: WOS:000403944700010.

545	42. Wilfling D, Dichter MN, Trutschel D, Kopke S. Prevalence of Sleep Disturbances in German

546	Nursing Home Residents with Dementia: A Multicenter Cross-Sectional Study. Journal of

547	Alzheimer's disease : JAD. 2019;69(1):227-36. Epub 2019/04/09. doi: 10.3233/jad-180784.

548	PubMed PMID: 30958349.

549	43. Gu DN, Sautter J, Pipkin R, Zeng Y. Sociodemographic and Health Correlates of Sleep Quality

550	and Duration among Very Old Chinese. Sleep. 2010;33(5):601-10. doi: 10.1093/sleep/33.5.601.

551	PubMed PMID: WOS:000277232200007.

552	44. Razali R, Ariffin J, Aziz AFA, Puteh SEW, Wahab S, Daud TIM. Sleep quality and psychosocial

553	correlates among elderly attendees of an urban primary care centre in Malaysia. Neurol Asia.

554	2016;21(3):265-73. PubMed PMID: WOS:000385421900008.

555 45. Yang CY, Chiou AF. Predictors of Sleep Quality in Community-Dwelling Older Adults in 556 Northern Taiwan. J Nurs Res. 2012;20(4):249-60. doi: 10.1097/jnr.0b013e3182736461. PubMed 557 PMID: WOS:000311276900003.

558 46. Mazzotti DR, Guindalini C, Sosa AL, Ferri CP, Tufik S. Prevalence and correlates for sleep

559 complaints in older adults in low and middle income countries: A 10/66 Dementia Research 560 Group study. Sleep medicine. 2012;13(6):697-702. doi: 10.1016/j.sleep.2012.02.009. PubMed 561 PMID: WOS:000306304700024.

562	47. Hayashino Y, Yamazaki S, Takegami M, Nakayama T, Sokejima S, Fukuhara S. Association

563	between number of comorbid conditions, depression, and sleep quality using the Pittsburgh

564	Sleep Quality Index: results from a population-based survey. Sleep medicine. 2010;11(4):366-71.

565	Epub 2010/03/12. doi: 10.1016/j.sleep.2009.05.021. PubMed PMID: 20219425.

566 48. Park JH, Yoo MS, Bae SH. Prevalence and predictors of poor sleep quality in Korean older 567 adults. Int	J	Nurs	Pract. 2013;19(2):116-23. doi: 10.1111/ijn.12047. PubMed	PMID: 568 WOS:000317675800002.

569	49. Yu J, Rawtaer I, Fam J, Jiang MJ, Feng L, Kua EH, et al. Sleep correlates of depression and

570	anxiety in an elderly Asian population. Psychogeriatrics. 2016;16(3):191-5. Epub 2015/07/17.

571 doi: 10.1111/psyg.12138. PubMed PMID: 26179204.

572 50. Paudel ML, Taylor BC, Diem SJ, Stone KL, Ancoli-Israel S, Redline S, et al. Association between

573 depressive symptoms and sleep disturbances in community-dwelling older men. Journal of the 574 American Geriatrics Society. 2008;56(7):1228-35. doi: 10.1111/j.1532-5415.2008.01753.x. 575 PubMed PMID: WOS:000258073800009.

576 51. Cho HJ, Lavretsky H, Olmstead R, Levin MJ, Oxman MN, Irwin MR. Sleep Disturbance and

577 Depression Recurrence in Community-Dwelling Older Adults: A Prospective Study. American 578 Journal of Psychiatry. 2008;165(12):1543-50. doi: 10.1176/appi.ajp.2008.07121882. PubMed 579 PMID: WOS:000261266000011.

580	52. Chang KJ, Son SJ, Lee Y, Back JH, Lee KS, Lee SJ, et al. Perceived sleep quality is associated

581	with depression in a Korean elderly population. Archives of Gerontology and Geriatrics.

582	2014;59(2):468-73.	doi:	10.1016/j.archger.2014.04.007.	PubMed	PMID:

583	WOS:000341018300038.

584	53. Potvin O, Lorrain D, Belleville G, Grenier S, Preville M. Subjective sleep characteristics

585	associated with anxiety and depression in older adults: a population-based study. International

586	journal of geriatric psychiatry. 2014;29(12):1262-70. doi: 10.1002/gps.4106. PubMed PMID:

587	WOS:000345214600010.

588 54. Press Y, Punchik B, Freud T. The association between subjectively impaired sleep and

589 symptoms of depression and anxiety in a frail elderly population. Aging Clinical and Experimental 590	Research. 2018;30(7):755-65. doi: 10.1007/s40520-017-0837-1. PubMed	PMID: 591 WOS:000435713200007.

592	55. Ahmed D, El Shair IH, Taher E, Zyada F. Prevalence and predictors of depression and anxiety

593	among the elderly population living in geriatric homes in Cairo, Egypt. The Journal of the

594	Egyptian	Public	Health	Association.	2014;89(3):127-35.	doi:

595	10.1097/01.epx.0000455729.66131.49. PubMed PMID: MEDLINE:25534177.

596	56. Kirmizioglu Y, Dogan O, Kugu N, Akyuz G. Prevalence of anxiety disorders among elderly

597	people. International journal of geriatric psychiatry. 2009;24(9):1026-33. doi: 10.1002/gps.2215.

598	PubMed PMID: WOS:000269428100017.

599 57. Teesson M, Mitchell PB, Deady M, Memedovic S, Slade T, Baillie A. Affective and anxiety 600 disorders and their relationship with chronic physical conditions in Australia: findings of the 601 2007 National Survey of Mental Health and Wellbeing. Aust N Z J Psych. 2011;45(11):939-46. doi: 602 10.3109/00048674.2011.614590. PubMed PMID: WOS:000296457400007.

603 58. Beekman ATF, de Beurs E, van Balkom A, Deeg DJH, van Dyck R, van Tilburg W. Anxiety and 604 depression in later life: Co-occurrence and communality of risk factors. American Journal of 605 Psychiatry. 2000;157(1):89-95. doi: 10.1176/ajp.157.1.89. PubMed PMID:

606 WOS:000084559900014.

607 59. Eshkoor SA, Hamid TA, Nudin SSH, Mun CY. The Effects of Social Support and Having a 608 Partner on Sleep Quality in Dementia. Am J Alzheimers Dis Other Dement. 2013;28(3):253-7. doi: 609 10.1177/1533317513481098. PubMed PMID: WOS:000318525500007.

610 60. Kishimoto Y, Okamoto N, Saeki K, Tomioka K, Obayashi K, Komatsu M, et al. Bodily pain, 611 social support, depression symptoms and stroke history are independently associated with sleep 612 disturbance among the elderly: a cross-sectional analysis of the Fujiwara-kyo study. Environ 613 Health Prev. 2016;21(5):295-303. doi: 10.1007/s12199-016-0529-z. PubMed PMID:

614 WOS:000381290400002.

615 61. Bazargan M. Self-reported sleep disturbance among African-American elderly: the effects of 616 depression, health status, exercise, and social support. International journal of aging & human 617	development. 1996;42(2):143-60. doi: 10.2190/gm89-nrty-derq-lc7d. PubMed	PMID: 618 MEDLINE:8859496.

619 62. Troxel WM, Buysse DJ, Monk TH, Begley A, Hall M. Does social support differentially affect 620 sleep in older adults with versus without insomnia? Journal of psychosomatic research. 621 2010;69(5):459-66. Epub 2010/10/20. doi: 10.1016/j.jpsychores.2010.04.003. PubMed PMID:

622 20955865; PubMed Central PMCID: PMCPMC2958100.

623 63. Olivera J, Benabarre S, Lorente T, Rodriguez M, Peleogrin C, Calvo JM, et al. Prevalence of 624 psychiatric symptoms and mental disorders detected in primary care in an elderly Spanish 625 population. The PSICOTARD Study: preliminary findings. International journal of geriatric 626 psychiatry. 2008;23(9):915-21. doi: 10.1002/gps.2004. PubMed PMID: WOS:000259148300006.

627 64. Paukert AL, Pettit JW, Kunik ME, Wilson N, Novy DM, Rhoades HM, et al. The Roles of Social 628 Support and Self-Efficacy in Physical Health's Impact on Depressive and Anxiety Symptoms in 629 Older Adults. J Clin Psychol Med Settings. 2010;17(4):387-400. doi: 10.1007/s10880-010-9211-6. 630 PubMed PMID: WOS:000285154000013.

631	65. Cacioppo JT, Hawkley LC. Social isolation and health, with an emphasis on underlying	632 mechanisms. Perspectives in biology and medicine. 2003;46(3 Suppl):S39-52. Epub 2003/10/18. 633 PubMed PMID: 14563073.

634 66. Costa SV, Ceolim MF, Neri AL. Sleep problems and social support: Frailty in a Brazilian 635 Elderly Multicenter study. Revista latino-americana de enfermagem. 2011;19(4):920-7. Epub 636 2011/08/31. doi: 10.1590/s0104-11692011000400010. PubMed PMID: 21876944.

637 67. Matsuda R, Kohno T, Kohsaka S, Fukuoka R, Maekawa Y, Sano M, et al. The prevalence of poor 638 sleep quality and its association with depression and anxiety scores in patients admitted for 639 cardiovascular disease: A cross-sectional designed study. Int J Cardiol. 2017;228:977-82. Epub 640 2016/12/05. doi: 10.1016/j.ijcard.2016.11.091. PubMed PMID: 27915216.

641 68. Bo Y, Yeoh EK, Guo C, Zhang Z, Tam T, Chan TC, et al. Sleep and the Risk of Chronic Kidney 642 Disease: A Cohort Study. Journal of clinical sleep medicine : JCSM : official publication of the 643 American	Academy	of	Sleep	Medicine. 2019;15(3):393-400. Epub	2019/03/12. doi: 644 10.5664/jcsm.7660. PubMed PMID: 30853043; PubMed Central PMCID: PMCPMC6411180.

645
