# Supplementary material for: The prevalence of poor sleep quality and associated risk factors among Chinese elderly adults in nursing homes: A cross-sectional study
Source: PLoS One. 2020 May 15;15(5):e0232834. doi: 10.1371/journal.pone.0232834 (PMC7228093; doi:10.1371/journal.pone.0232834)
Supplement: Attachment — Submitted filename: Response to Reviewers.docx [file pone.0232834.s002.docx]

Response to [PONE-D-19-31759]

Thank you very much for your valuable comments and critiques, which have improved the clarity of our manuscript to a greater extent. Accordingly, we have thoroughly revised our manuscript based on the editors’ and reviewers’ comments and suggestions. The changes are highlighted in red for added words, or strikethrough for deleted words. Additionally, minor language, grammatical and stylistic errors have been corrected. Our specific point-by-point responses to the comments and queries have been addressed and itemized as following:

Journal Requirements:

When submitting your revision, we need you to address these additional requirements.

1. Please ensure that your manuscript meets PLOS ONE's style requirements, including those for file naming. The PLOS ONE style templates can be found at

<http://www.journals.plos.org/plosone/s/file?id=wjVg/PLOSOne_formatting_sample_main_body.pdf> and <http://www.journals.plos.org/plosone/s/file?id=ba62/PLOSOne_formatting_sample_title_authors_affiliations.pdf>

**Answer:** Thank you so much for your helpful comment. We have checked and ensured that our manuscript meets PLOS ONE’s style requirements.

2. Please provide additional details regarding participant consent. In the ethics statement in the Methods and online submission information, please ensure that you have specified (1) whether consent was suitably informed and (2) what type you obtained (for instance, written or verbal). If your study included minors under age 18, state whether you obtained consent from parents or guardians. If the need for consent was waived by the ethics committee, please include this information.

**Answer:** Thank you so much for your valuable comment. We have included the ethics statement in the Methods section as follows: *This study was approved by the Ethics Committee of Xiangya School of Public Health of Central South University (No.XYGW-2018-49). Written informed consent was obtained from all participants of this study.*

3. Please include additional information regarding the survey or questionnaire used in the study and ensure that you have provided sufficient details that others could replicate the analyses. If you developed and/or translated a questionnaire as part of this study and it is not under a copyright more restrictive than CC-BY, please include a copy, in both the original language and English, as Supporting Information.

**Answer:** Thank you so much for your valuable comment. As suggested, we have included additional information regarding the survey or questionnaires used in the study. However, we did not develop nor translate a questionnaire as part of this study. Actually, all the questionnaires used in this study are Chinese versions, which have good reliability and validity as indicated in previous publish related studies.

4. We note that you have indicated that data from this study are available upon request. PLOS only allows data to be available upon request if there are legal or ethical restrictions on sharing data publicly. For information on unacceptable data access restrictions, please see http://journals.plos.org/plosone/s/data-availability#loc-unacceptable-data-access-restrictions.

**Answer:** Thank you so much for this important information. We have since checked on this link about information on unacceptable data access restrictions.

In your revised cover letter, please address the following prompts:

a) If there are ethical or legal restrictions on sharing a de-identified data set, please explain them in detail (e.g., data contain potentially identifying or sensitive patient information) and who has imposed them (e.g., an ethics committee). Please also provide contact information for a data access committee, ethics committee, or other institutional body to which data requests may be sent.

b) If there are no restrictions, please upload the minimal anonymized data set necessary to replicate your study findings as either Supporting Information files or to a stable, public repository and provide us with the relevant URLs, DOIs, or accession numbers. Please see http://www.bmj.com/content/340/bmj.c181.long for guidelines on how to de-identify and prepare clinical data for publication. For a list of acceptable repositories, please see http://journals.plos.org/plosone/s/data-availability#loc-recommended-repositories.

We will update your Data Availability statement on your behalf to reflect the information you provide.

**Answer:** Thank you so much for your valuable comments and suggestions. We provided our data availability statement as follows: *Data for this study contain potentially identifying or sensitive patient information. Therefore, it would be available upon reasonable request from the Ethics Committee of Xiangya School of Public Health of Central South University (csugwxy@126.com).*

5. PLOS requires an ORCID iD for the corresponding author in Editorial Manager on papers submitted after December 6th, 2016. Please ensure that you have an ORCID iD and that it is validated in Editorial Manager. To do this, go to ‘Update my Information’ (in the upper left-hand corner of the main menu), and click on the Fetch/Validate link next to the ORCID field. This will take you to the ORCID site and allow you to create a new iD or authenticate a pre-existing iD in Editorial Manager. Please see the following video for instructions on linking an ORCID iD to your Editorial Manager account: <https://www.youtube.com/watch?v=_xcclfuvtxQ>

**Answer:** Thank you so much for this important information. Accordingly, we have linked an ORCID ID for the corresponding author.

6. Your ethics statement must appear in the Methods section of your manuscript. If your ethics statement is written in any section besides the Methods, please move it to the Methods section and delete it from any other section. Please also ensure that your ethics statement is included in your manuscript, as the ethics section of your online submission will not be published alongside your manuscript.

**Answer:** Thank you so much for your valuable comment and suggestion. We have included our ethics statement in the Methods section as follows: *This study was approved by the Ethics Committee of Xiangya School of Public Health of Central South University (No.XYGW-2018-49). Written informed consent was obtained from all participants of this study.*

**Comments to the Author**

1. Is the manuscript technically sound, and do the data support the conclusions?

The manuscript must describe a technically sound piece of scientific research with data that supports the conclusions. Experiments must have been conducted rigorously, with appropriate controls, replication, and sample sizes. The conclusions must be drawn appropriately based on the data presented.

Reviewer #1: Yes

Reviewer #2: Yes

**Answer:** Thank you so much for being satisfied with the technical level of our manuscript.

2. Has the statistical analysis been performed appropriately and rigorously?

Reviewer #1: Yes

Reviewer #2: Yes

**Answer:** Thank you so much for your satisfaction with the statistical analysis in our manuscript.

3. Have the authors made all data underlying the findings in their manuscript fully available?

The PLOS Data policy requires authors to make all data underlying the findings described in their manuscript fully available without restriction, with rare exception (please refer to the Data Availability Statement in the manuscript PDF file). The data should be provided as part of the manuscript or its supporting information, or deposited to a public repository. For example, in addition to summary statistics, the data points behind means, medians and variance measures should be available. If there are restrictions on publicly sharing data—e.g. participant privacy or use of data from a third party—those must be specified.

Reviewer #1: Yes

Reviewer #2: Yes

**Answer:** Thank you so much for verifying that we provided the data availability statement as regards our manuscript.

4. Is the manuscript presented in an intelligible fashion and written in standard English?

PLOS ONE does not copyedit accepted manuscripts, so the language in submitted articles must be clear, correct, and unambiguous. Any typographical or grammatical errors should be corrected at revision, so please note any specific errors here.

Reviewer #1: Yes

Reviewer #2: Yes

**Answer:** Thank you so much for your satisfaction with the way our manuscript has been presented.

5. Review Comments to the Author

Please use the space provided to explain your answers to the questions above. You may also include additional comments for the author, including concerns about dual publication, research ethics, or publication ethics. (Please upload your review as an attachment if it exceeds 20,000 characters)

Reviewer #1: This study presents the original research that meets all applicable standard in ethics. Statistics, and other analyses are describe in sufficient detail. This article using standard English. Conclusions are presented and are supported by the data.

**Answer:** Thank you so much for approving the importance and quality of our study.

Reviewer #2: I agree that sleep problems have become the most common complaints among elderly adults and I believe the study presented by the Authors from China are important. In addition to that the study sample is large and therefore the correlations shown beetwen the variables are strong.

In my opinion it is a well written study. I only wonder why the Authors have chosen the eldrely with 60 age and above not from 65? and how did you judge the cognitive problesm as it is mentioned in the exculsion criteria?

**Answer:** Thank you so much for approving the importance and quality of our study. Also, thank you so much for your constructive questions regarding the sample selection and identification of cognitive problems in our manuscript. In China, the elderly age standard starting point is 60 years, which was provided by <Law of the People's Republic of China on Protection of the Rights and Interests of the Elderly> and National Bureau of Statistics of China. In addition, we have excluded participants who had a history of severe cognitive deficit based on the disease archives information provided by caregivers or health service providers in the respective nursing homes.
