# Supplementary material for: The prevalence of poor sleep quality and associated risk factors among Chinese elderly adults in nursing homes: A cross-sectional study
Source: PLoS One. 2020 May 15;15(5):e0232834. doi: 10.1371/journal.pone.0232834 (PMC7228093; doi:10.1371/journal.pone.0232834)
Supplement: Attachment — Submitted filename: REVIEW PONE-D EE.docx [file pone.0232834.s003.docx]

**The prevalence of poor sleep quality and associated risk factors among Chinese elderly adults in nursing homes: a cross-sectional study**

Xidi Zhu ^1¶^, Zhao Hu ^1¶^, Yu Nie^1^, Tingting Zhu^2^,Yunhan Yu^1^, Huilan Xu^1,*^

^1^ Department of Social Medicine and Health Management, Xiangya School of Public Health, Central South University, Changsha, China

^2^ Department of Scientific Research Management, Shanghai Health Development Research Center, Shanghai, China

*Corresponding author

E-mail address:xhl6363@sina.com

¶ These authors contributed equally to this work

**Abstract**

**Background:** Sleep problems have become the most common complaints among elderly adults. There are a few studies indentified prevalence of poor sleep quality and its associated factors in nursing home setting.Therefore, our study aim to examine the prevalence of poor sleep quality, its risk factors, and their interactions among Chinese elderly adults in nursing homes.

**Methods:** A total of 817 elderly residents from 24 nursing homes were included in this cross-sectional study. Sleep quality was assessed using the Pittsburgh Sleep Quality Index (PSQI), and poor sleep quality was defined as PSQI >5. Multiple binary logistic regression was used to estimate adjusted odds ratios (AORs) and 95% confidence intervals (CIs) between risk factors and poor sleep quality. An additional interaction model was used to analyse the interaction between risk factors.

**Results:** The prevalence of poor sleep quality was 67.3% (95% CI: 64.0, 70.5%) among elderly adults in nursing homes. Multiple binary logistic regression results showed that participants with the following characteristics had an increased risk of poor sleep quality after adjustments for other confounders: being 70-79 years old (AOR: 1.81, 95% CI: 1.10, 2.97) or 80 years old and above (AOR: 2.64, 95% CI: 1.67, 4.17); having less than 7 years of education (AOR: 1.59, 95% CI: 1.08, 2.33); having one to two kinds of chronic diseases (AOR: 2.26, 95% CI: 1.53, 3.32) or three or more kinds of chronic disease (AOR: 2.81, 95% CI: 1.65, 4.76); having depression symptoms (AOR: 3.13, 95% CI: 2.04, 4.81), anxiety symptoms (AOR: 3.42, 95% CI: 1.68, 6.97), and lower social support (AOR: 1.57, 95% CI: 1.11, 2.21). Additive interactions were detected between age and anxiety symptoms (AOR: 8.34, 95% CI: 4.43, 15.69), between chronic disease and anxiety symptoms (AOR: 8.61, 95% CI; 4.28, 17.31) and between social support and anxiety symptoms (AOR: 6.43, 95% CI: 3.22, 12.86).

**Conclusions**: The prevalence of poor sleep quality in nursing homes is relatively high. Anxiety symptoms has additive interactions with age, chronic disease and social support for poor sleep quality. These findings have significant implications for interventions that aim to improve sleep quality among elderly residents in nursing homes.

**Keywords:** sleep quality; nursing home; interaction; risk factors

**Introduction**

Ageing is a severe problem in China as well as throughout the world. According to the data of the Sixth National Population Census in 2010, the number of people in China aged 60 years and above was approximately 177 million, accounting for 13.26% of the total population[1]. The U.N. Commission On Population Development has predicted that the elderly population aged 60 and above in China will reach 243.8 million by 2020, accounting for 17.1% of the total population [2]. Sleep problems have become the most common complaints among elderly adults as they age. Approximately 74% of elderly men and 79% of elderly women reported sleep complaints in Italy[3]. In addition, studies have reported that the prevalence of sleep disorders was approximately 30-40% among older residents[4, 5]. However, as a complicated phenomenon, the sleep quality of individuals is difficult to define and assess objectively. Using a standardized assessment tool for sleep quality named the Pittsburgh Sleep Quality Index (PSQI), epidemiological studies have indicated that the self-rated prevalence of poor sleep quality was 62.4% among older adults in Thailand[6], 16.6% in Mexican Americans aged 75 and older[7], 41.5% and 33.8% among elderly adults living in urban and rural areas in China, respectively[8, 9]. However, several studies demonstrated that most sleep problems may be exacerbated by institutional setting[10, 11]. For instance, a study conducted among 100 selected individuals over 65 years of age found that institutionalized elderly adults presented worse overall sleep quality and higher levels of daytime somnolence than non-institutionalized elderly adults[10]. Fetveit and colleagues [11] found that the prevalence of sleep disturbance was approximately 70% among nursing home residents. In summary, sleep problems are very common among elderly adults and pose a major challenge to public health.

Evidence is emerging that poor sleep quality is positively associated with physical, mental disorders and quality of life[12, 13]. A large cohort study found that poor sleep quality was associated with a higher odds of hypertension in a Chinese rural population[13]. Another large cohort study indicated that short sleepers with poor sleep quality had an increased risk of cardiovascular disease[14]. In addition, many studies[15-17] have indicated that poor sleep quality is a powerful predictor of suicidal ideation and depressive symptoms among elderly adults. However, the underlying mechanisms between poor sleep quality and these harmful consequences are still unclear.

To alleviate the personal suffering and adverse effects introduced by poor sleep quality, it is essential to understand its prevalence pattern and associated factors. Previous studies have demonstrated many factors related to poor sleep quality among older adults, including but not limited to the following four domains: (1) socio-demographic factors, such as age[9], marital status[18] and education[19]; (2) lifestyle factors, such as physical activity [20] and caffeine intake[21]; (3) emotional factors, such as stress[22] and depression[20]; and (4) chronic conditions, such as arthritis[9] and pulmonary disease[23]. However, few studies have examined the prevalence and risk factors of poor sleep quality in a nursing home population. Moreover, no study has explored the interactions of risk factors for poor sleep quality among elderly residents in nursing homes. Thus, the purposes of our study were as follows: (1) to examine the prevalence of poor sleep quality among elderly adults in nursing homes in China; (2) to analyse the risk factors for poor sleep quality; and (3) to explore the interactions of risk factors for poor sleep quality.

**Materials and methods**

**Study population**

This was a cross-sectional study conducted in nursing homes in Hunan Province in China from October to December 2018. This study was approved by the Ethical Committee of Xiangya School of Public Health in Central South University (No.XYGW-2018-49).Written informed consent was obtained from all participants of this study. To select a representative elderly sample living in nursing homes of Hunan Province, a multistage sampling method was used. First, each city from northern Hunan, southern Hunan and central Hunan was selected (i.e., based on geographical region): Changsha city, Hengyang city and Yiyang city, respectively. Subsequently, one county was randomly chosen from each selected city: Changsha County, Hengyang County and Yuanjiang County. Second, two districts (Kaifu and Yuelu) from Changsha city and two townships (Xingsha and Tiaoma) from Changsha County were randomly selected. Similarly, Yanfeng and Shigu from Hengyang city and Xidu and Jingtou from Hengyang County were randomly selected. Ziyang and Heshan from Yiyang city and Qionghu and Caowei from Yuanjiang County were randomly chosen. Finally, two nursing homes were randomly selected from each selected district and township, and a total of 24 nursing homes were ultimately selected.

Resident populations in selected nursing homes were included in our study if they met the following inclusion criteria: (1) age 60 years and above; (2) duration of entrance into nursing homes of more than one year; and (3) physical and mental ability to participate in interviews. Participants were excluded if they (1) refused to participate in this study, (2) had a severe hearing impairment or language barrier, and (3) had a history of severe cognitive deficit. In summary, among a total of 2,055 older adults residents in the 24 nursing homes, 511 adults were excluded because they were younger than 60 years old or had stayed in a nursing home less than one year. A total of 603 older adults had a severe hearing impairment, language barrier or severe cognitive deficit, and 112 older adult residents who did not agree to participate were also excluded from this study. Of the remaining 829 older adults, 12 were excluded due to incomplete data. Ultimately, a total of 817 elderly adults were included in the data analysis in this study.

**Data measurement**

All data were obtained through face-to-face interviews by trained staff who spent 30 minutes to 1 hour following a structured self-administrate questionnaire. We collected data on socio-demographics (age, gender, education, marital status, monthly individual income, number of descendants, length of stay in a nursing home and medical insurance status), lifestyle behavioural factors (smoking, alcohol drinking and physical exercise), physical status (activities of daily living (ADLs) and number of chronic disease) and social psychological factors (social support, depression symptoms and anxiety symptoms). Marital status was dichotomized into stable and unstable. Unstable marital status included divorce, the loss of a partner and never having been married. Smoking was assessed by a single item and defined as an average of at least one cigarette per day in the last year. Alcohol drinking was assessed by a single item and defined as drinking a glass of wine per day in the last week. Regular physical exercise refers to engaging in at least 30 minutes of physical exercise at least three times a week. ADL status was assessed using Chinese version of Lawton and Brody’s ADL scale, which subsequently dichotomized participants into disabled and normal[24]. Disabled ADL status was determined when participants had a total score of greater than 14 points. Cronbach’s α was 0.926 in this study. Self-reported chronic diseases were assessed by a multiple choice question, which refer to chronic non-communicable diseases, including hypertension, diabetes, coronary heart disease (CHD), chronic obstructive pulmonary disease (COPD), hyperlipidaemia, stroke and others. Social support was examined using the Social Support Rating Scale (SSRS), which was developed by Xiao and is widely used in China. Then, participants were divided into two groups based on the median of the global score (lower: score≤30 points; higher: score>30 points). Depression symptoms were assessed using the Chinese version of the Geriatric Depression Scale-30 items (GDS-30) [25], which indicated high validity and reliability among the Chinese elderly population[26]. Cronbach’s α was 0.925 in this study. The GDS-30 consists of 30 true/false questions, and the total score ranges from 0 to 30 points. Participants who scored 11 and above were considered to have depression symptoms in this study. Anxiety symptoms were assessed using the Chinese version of the Generalized Anxiety Disorder-7 scale (GAD-7). The GAD-7 is a seven-item self-report instrument, and each item assesses one of the typical symptoms of GAD over the last two weeks, with a total score that ranges from 0 to 21. Cronbach’s α was 0.933 in this study.Participants who scored 10 and above were considered to have anxiety symptoms in this study [27].

**Assessment of sleep quality**

Sleep quality among elderly residents in nursing homes was assessed using the Chinese version of the Pittsburgh Sleep Quality Index (PSQI)[28], which has been proven to have good validity and reliability among older adults[29]. The PSQI is a self-rated questionnaire that assesses sleep quality in the past month. It contains nineteen items grouped into seven components: subjective sleep quality, sleep latency, sleep duration, sleep efficiency, sleep disturbances, use of sleeping medication, and daytime dysfunction. Each component score ranges from 0 to 3, and the total score ranges from 0 to 21 points. Participants with a global PSQI score greater than 5 were defined as having poor sleep quality in this study. Cronbach’s α was 0.781 in this study.

**Statistical analysis**

Categorical variables are presented as frequencies and percentages. Continuous variables are presented as the mean±standard deviation (SD). The differences between continuous variables were compared using Student’s t-test or one-way analysis of variance (ANOVA). The differences between categorical variables were analysed using the Pearson chi-square test or the Mantel-Haenszel chi-square test. Multiple binary logistic regression analysis was used to examine risk factors associated with poor sleep quality. The adjusted odds ratios (AORs) and 95% confidence intervals (CIs) were also estimated after controlling for other confounders. Microsoft Excel, developed by Andersson and colleagues[30], was used to detect and calculate the additive interaction of risk factors for poor sleep quality. The relative excess risk due to interaction (RERI) and the attributable proportion due to interaction (AP) being equal to 0 or the synergy index (S) being equal to 1 suggest no additive interaction between the two factors. *P*<0.05 was considered to be statistically significant. All statistical analyses were analysed using SAS 9.3 (SAS Institute Inc., Cary, NC, USA).

**Results**

**Characteristics of the study sample**

In total, 817 elderly residents living in nursing homes were included in this study. Among them, more than half of participants aged 80 years and above. A total of 54.0% of subjects were female, and 46.0% were male. Approximately 40% of the participants had completed less than 6 years of education and had stable marriages. Approximately one-third reported monthly individual incomes of more than 3,000 RMB. A minority of participants engaged in cigarette smoking and alcohol drinking and had no chronic diseases. Approximately 36.0% of individuals had depressive symptoms, and 16.0% had anxiety symptoms.

There were significant statistically significant differences between poor sleepers and good sleepers in age, education, marital status, monthly individual income, length of stay, number of chronic diseases, depression symptoms, anxiety symptoms, social support and ADL status (all *P*<0.05). Poor sleepers were seemingly those who were older in age, had lower education, had unstable marriages, had lower monthly individual incomes, had longer lengths of stay, had at least one type of chronic disease, had depression symptoms, had anxiety symptoms, had lower social support and had disabled ADLs. The results are presented in Table 1.

Table 1 Characteristics of elderly residents in nursing homes in this study

| Characteristic | Total  (n=817) | Poor sleeper  (n=550) | Good sleeper  (n=267) | *P*-value^†^ |
| --- | --- | --- | --- | --- |
| Age (years) |  |  |  |  |
| 60~ | 135(16.5) | 73(13.3) | 62(23.2) | <0.001 |
| 70~ | 232(28.4) | 150(27.3) | 82(30.7) |  |
| 80~ | 450(55.1) | 327(59.4) | 123(46.1) |  |
| Gender |  |  |  |  |
| Male | 376(46.0) | 251(45.6) | 125(46.8) | 0.751 |
| Female | 441(54.0) | 299(54.4) | 142(53.2) |  |
| Education (years) |  |  |  |  |
| 0-6 | 364(44.6) | 263(47.8) | 101(37.8) | 0.004 |
| 7~ | 203(24.8) | 138(25.1) | 65(24.4) |  |
| 10~ | 250(30.6) | 149(27.1) | 101(37.8) |  |
| Marital status |  |  |  |  |
| Stable | 302(37.0) | 186(33.8) | 116(43.4) | 0.007 |
| Unstable | 515(63.0) | 364(66.2) | 151(56.6) |  |
| Medical insurance |  |  |  |  |
| Yes | 766(93.8) | 517(94.0) | 249(93.3) | 0.681 |
| No | 51(6.2) | 33(6.0) | 18(6.7) |  |
| Monthly individual income |  |  |  |  |
| ≤3,000 RMB | 568(69.5) | 404(73.5) | 164(61.4) | <0.001 |
| >3,000 RMB | 249(30.5) | 146(26.5) | 103(38.6) |  |
| Length of stay (years) |  |  |  |  |
| 1~ | 444(54.3) | 284(51.6) | 160(59.9) | 0.026 |
| 3~ | 373(45.7) | 266(48.4) | 107(40.1) |  |
| Have descendants |  |  |  |  |
| Yes | 746(91.3) | 496(90.2) | 250(93.6) | 0.100 |
| No | 71(8.7) | 54(9.8) | 17(6.4) |  |
| Smoking |  |  |  |  |
| Yes | 132(16.2) | 82(14.9) | 50(18.7) | 0.164 |
| No | 685(83.8) | 468(85.1) | 217(81.3) |  |
| Alcohol drinking |  |  |  |  |
| Yes | 96(11.8) | 60(10.9) | 36(13.5) | 0.284 |
| No | 721(88.2) | 490(89.1) | 231(76.5) |  |
| Physical exercise |  |  |  |  |
| Regular | 353(43.2) | 233(42.4) | 120(44.9) | 0.485 |
| Irregular | 464(56.8) | 317(57.6) | 147(55.1) |  |
| Number of chronic diseases |  |  |  |  |
| 0 | 176(21.5) | 86(15.6) | 90(33.7) | <0.001 |
| 1~ | 471(57.7) | 328(59.7) | 143(53.6) |  |
| 3~ | 170(20.8) | 136(24.7) | 34(12.7) |  |
| ADL status |  |  |  |  |
| Normal | 268(32.8) | 152(27.6) | 116(43.4) | <0.001 |
| Disabled | 549(67.2) | 398(72.4) | 151(56.6) |  |
| Depression symptoms |  |  |  |  |
| Yes | 294(36.0) | 251(45.6) | 43(16.1) | <0.001 |
| No | 523(64.0) | 299(54.4) | 224(83.9) |  |
| Anxiety symptoms |  |  |  |  |
| Yes | 136(16.6) | 124(22.5) | 12(4.5) | <0.001 |
| No | 681(83.4) | 426(77.5) | 255(95.5) |  |
| Social support |  |  |  |  |
| Low | 435(53.2) | 337(61.3) | 98(36.7) | <0.001 |
| High | 382(46.8) | 213(38.7) | 169(63.3) |  |

Data are presented as n(%). ADLs, activities of daily living.

† P-value was determined by the Pearson chi-square test.

**Prevalence of poor sleep quality**

The mean global PSQI score was 8.5 points overall (95% CI: 8.2, 8.8), 8.3 points in males (95% CI: 7.8, 8.8) and 8.7 points in females (95% CI: 8.2, 9.1). There was no statistically significant difference in the PSQI score between males and females (*P*=0.293). The total PSQI score increased with increasing age, from 7.4 points (95% CI: 6.6, 8.2) in subjects aged 60-69 years to 8.9 points (95% CI: 8.4, 9.3) in subjects aged ≥80 years (*P* for trend=0.010). A total of 550 individuals had a total PSQI score greater than 5, and the overall prevalence of poor sleep quality was 67.3% (95% CI: 64.0%, 70.5%). The prevalence of poor sleep quality was 66.8% (95% CI: 61.7%, 71.0%) in males and 67.8% (95% CI: 63.5%, 72.1%) in females. The prevalence rate was also increased by increasing age, ranging from 54.1% (95% CI: 45.9%, 63.0%) in participants aged 60-69 years to 72.7% (95% CI: 68.7%, 77.1%) in participants aged ≥80 years (*P* for trend <0.001). The results are shown in Table 2.

Table 2 The PSQI global score and prevalence of poor sleep quality in nursing homes stratified by age and gender

|  | N | PSQI score  Mean(95% CI) | P-value |  | Poor sleep quality^†^  Prevalence(95% CI) | P-value |
| --- | --- | --- | --- | --- | --- | --- |
| Total | 817 | 8.5(8.2, 8.8) |  |  | 67.3(64.0, 70.5) |  |
| Gender |  |  |  |  |  |  |
| Male | 376 | 8.3(7.8, 8.8) | 0.293 |  | 66.8(61.7, 71.0) | 0.751 |
| Female | 441 | 8.7(8.2, 9.1) |  |  | 67.8(63.5, 72.1) |  |
| Age |  |  |  |  |  |  |
| 60~ | 135 | 7.4(6.6, 8.2) | 0.010^*^ |  | 54.1(45.9, 63.0) | <0.001^*^ |
| 70~ | 232 | 8.4(7.8, 9.1) |  |  | 64.7(58.6, 70.5) |  |
| 80~ | 450 | 8.9(8.4, 9.3) |  |  | 72.7(68.7, 77.1) |  |

†Poor sleep quality was determined by PSQI score >5.

**P*-value for trend.

**Components of sleep quality**

Most of the participants went to bed at 9 PM and woke up at approximately 6 AM. The average bedtime of the participants was 9.1±3.5 hours, and the average sleep duration was 6.8±3.9 hours. Most subjects could not fall asleep within 30 minutes. The average sleep efficiency of the participants was 80.0%. Approximately 85% of subjects never used sleep medication. The mean scores for subjective sleep quality, sleep disturbance and daytime dysfunction were 1.3±0.9, 1.2±0.6 and 1.4 ±1.1, respectively.

No statistically significant differences were observed between males and females on all components of sleep quality. More older residents than younger people have used sleep medication. Moreover, older adults have more daytime dysfunction than younger individuals do. No significant differences were obtained in sleep latency, sleep efficiency or sleep disturbance between age groups. The results are shown in Table 3.

Table 3 Gender- and age-specific scores of sleep quality components measured by the Chinese version of the Pittsburgh Sleep Quality Index

| Sleep quality components |  | Total  (n=817) | Gender | | |  | Age group (years) | | | |
| --- | --- | --- | --- | --- | --- | --- | --- | --- | --- | --- |
|  |  |  | Male  (n=376) | Female  (n=441) | P-value |  | 60-69  (n=135) | 70-79  (n=232) | ≥80  (n=450) | P-value |
| Subjective sleep quality^†^, mean±SD |  | 1.3±0.9 | 1.3±0.9 | 1.3±0.9 | 0.674 |  | 1.2±0.9 | 1.3±0.9 | 1.3±0.9 | 0.482 |
| Sleep latency, n(%) | ≤15 min | 236(28.9) | 116(30.9) | 120(27.2) | 0.200 |  | 51(37.8) | 63(27.2) | 122(27.1) | 0.161 |
|  | 16~min | 252(30.8) | 121(32.2) | 131(29.7) |  |  | 34(25.2) | 75(32.3) | 143(31.8) |  |
|  | 31~min | 329(40.3) | 139(36.9) | 190(43.1) |  |  | 50(37.0) | 94(40.5) | 185(41.1) |  |
| Sleep duration, mean±SD |  | 6.8±3.9 | 6.8±3.1 | 6.8±4.4 | 0.908 |  | 6.9±2.1 | 6.7±2.7 | 6.9±4.7 | 0.759 |
| Sleep duration, n(%) | <6 h | 262(32.1) | 121(32.2) | 141(32.0) | 0.253 |  | 38(28.1) | 80(34.5) | 144(32.0) | 0.008 |
|  | 6-7 h | 278(34.0) | 118(31.4) | 160(36.3) |  |  | 38(28.1) | 67(28.9) | 173(38.4) |  |
|  | >7 h | 277(33.9) | 137(36.4) | 140(31.7) |  |  | 59(43.8) | 85(36.6) | 133(29.6) |  |
| Habitual sleep efficiency^*^, mean±SD |  | 0.8±0.4 | 0.8±0.3 | 0.8±0.4 | 0.593 |  | 0.8±0.2 | 0.7±0.2 | 0.8±0.5 | 0.558 |
| Habitual sleep efficiency, n(%) | <65% | 270(33.0) | 125(33.2) | 145(32.8) | 0.803 |  | 41(30.4) | 80(34.5) | 149(33.1) | 0.075 |
|  | 65-74% | 103(12.6) | 48(12.8) | 55(12.5) |  |  | 14(10.4) | 28(12.1) | 61(13.6) |  |
|  | 75-84% | 135(16.5) | 57(15.2) | 78(17.7) |  |  | 15(11.1) | 34(14.7) | 86(19.1) |  |
|  | >85% | 309(37.8) | 146(38.8) | 163(37.0) |  |  | 65(48.1) | 90(38.8) | 154 (34.2) |  |
| Sleep disturbances^‡^, mean±SD |  | 1.2±0.6 | 1.2±0.6 | 1.2±0.6 | 0.914 |  | 1.2±0.6 | 1.3±0.6 | 1.3±0.5 | 0.148 |
| Any sleep disturbance, n(%) | Yes | 248(30.4) | 112(29.8) | 136(30.8) | 0.745 |  | 38(28.1) | 72(31.0) | 138(30.7) | 0.826 |
|  | No | 569(69.6) | 264(60.2) | 305(69.2) |  |  | 97(71.9) | 160(69.0) | 312(69.3) |  |
| Use of sleep medication, n(%) | Yes | 115(14.1) | 44(11.7) | 71(16.1) | 0.072 |  | 9(6.7) | 23(9.9) | 83(18.4) | <0.001 |
|  | No | 702(85.9) | 332(88.3) | 370(83.9) |  |  | 126(93.3) | 209(90.1) | 367(81.6) |  |
| Daytime dysfunction^**^, mean±SD |  | 1.4±1.1 | 1.4±1.1 | 1.5±1.0 | 0.620 |  | 1.2 ±1.0 | 1.5±1.1 | 1.5±1.0 | 0.037 |

SD, standard deviation. †Score range 0 to 3; higher scores indicate poor subjective sleep quality;*Derived from the formula: hours of sleep/(get-up time−usual bedtime)×100%; score range from 0 to 1; higher scores indicate higher sleep efficiency; ‡Derived from PSQI items 5b-5j; score range from 0 to 3; higher scores indicate more sleep disturbances;**Derived from PSQI item 8 and 9; score range from 0 to 3; higher scores indicate more daytime dysfunction.

**Risk factors for poor sleep quality**

Multivariate binary logistic regression analysis results suggest that age between 70 to 79 years (AOR: 1.81, 95% CI: 1.10, 2.97) or 80 years and above (AOR: 2.64, 95% CI: 1.67, 4.17) increased the risk of poor sleep quality compared to age between 60 and 69 years after adjustments were made for other confounding factors. Moreover, similar results also indicated that participants with 0-6 years of education (AOR: 1.59, 95% CI: 1.08, 2.33), one to two kinds of chronic diseases (AOR: 2.26, 95% CI: 1.53, 3.32) or three or more kinds of chronic diseases (AOR: 2.81, 95%: 1.65, 4.76), depression symptoms (AOR: 3.13, 95% CI: 2.04, 4.81) or anxiety symptoms (AOR: 3.42, 95% CI: 1.68, 6.97) and lower social support (AOR: 1.57, 95% CI: 1.11, 2.21) were more likely to report poor sleep quality after adjustments were made for other variables. The results are shown in Table 4.

Table 4 Risk factors associated with poor sleep quality among elderly adults in nursing homes

| Variable | B | SE | Wald | AOR(95% CI)^†^ | P-value |
| --- | --- | --- | --- | --- | --- |
| Age (years) |  |  |  |  |  |
| 60~ |  |  |  | 1.00 |  |
| 70~ | 0.59 | 0.25 | 5.51 | 1.81(1.10, 2.97) | 0.019 |
| 80~ | 0.97 | 0.23 | 17.32 | 2.64(1.67, 4.17) | <0.001 |
| Education (years) |  |  |  |  |  |
| 10~ |  |  |  | 1.00 |  |
| 7~ | 0.34 | 0.22 | 2.28 | 1.40(0.90, 2.16) | 0.132 |
| 0-6 | 0.46 | 0.20 | 5.49 | 1.59(1.08, 2.33) | 0.019 |
| Number of chronic diseases |  |  |  |  |  |
| 0~ |  |  |  | 1.00 |  |
| 1~ | 0.82 | 0.20 | 17.02 | 2.26(1.53, 3.32) | <0.001 |
| 3~ | 1.03 | 0.27 | 14.61 | 2.81(1.65, 4.76) | <0.001 |
| Depression symptoms |  |  |  |  |  |
| No |  |  |  | 1.00 |  |
| Yes | 1.14 | 0.22 | 27.05 | 3.13(2.04, 4.81) | <0.001 |
| Anxiety symptoms |  |  |  |  |  |
| No |  |  |  | 1.00 |  |
| Yes | 1.23 | 0.36 | 11.53 | 3.42(1.68, 6.97) | 0.001 |
| Social support |  |  |  |  |  |
| High |  |  |  | 1.00 |  |
| Low | 0.45 | 0.18 | 6.51 | 1.57(1.11, 2.21) | 0.011 |

SE, standard error; AOR, adjusted odds ratio.

†Adjusted for gender, marital status, smoking, alcohol drinking, physical exercise, descendants, medical insurance, monthly individual income, length of stay and ADL status, as well as the variables in the table.

**Interactions of risk factors for poor sleep quality**

In this study, Andersson’s Excel was used to detect the interactions and calculate the effect size between risk factors for poor sleep quality. The adjusted factors included all variables (except for two), the interactions among which we would examine. Additional interactions were detected between the following variables: older age and anxiety symptoms, with an RERI of 2.83 (95% CI: 0.13, 5.52), an AP of 0.34 (95% CI: 0.13, 0.55) and an S of 1.63 (95% CI: 1.11, 2.39); chronic disease and anxiety symptoms, with an RERI of 3.32 (95% CI: 0.31, 6.33), an AP of 0.39 (95% CI: 0.29, 0.48) and an S of 1.77 (95% CI: 1.54, 2.04); and social support and anxiety symptoms, with an RERI of 2.96 (95% CI: 1.02, 4.90), an AP of 0.46 (95% CI: 0.38, 0.54) and an S of 2.20 (95% CI: 1.65, 2.93). No statistically significant interactions were found between other factors after adjusting for confounders. The results are shown in Table 5.

Table 5 Interactions of risk factors for poor sleep quality among elderly adults in nursing homes

| Factor 1 | Factor 2 | AOR^†^ | RERI | AP | S |
| --- | --- | --- | --- | --- | --- |
| Age (years) | Anxiety symptoms |  | 2.83(0.13, 5.52) | 0.34(0.13, 0.55) | 1.63(1.11, 2.39) |
| 60-69 | No | 1.00 |  |  |  |
| ≥70 | No | 2.09(1.01, 4.32) |  |  |  |
| 60-69 | Yes | 4.43(2.13, 9.20) |  |  |  |
| ≥70 | Yes | 8.34(4.43, 15.69) |  |  |  |
| Chronic disease | Anxiety symptoms |  | 3.32(0.31, 6.33) | 0.39(0.29, 0.48) | 1.77(1.54, 2.04) |
| No | No | 1.00 |  |  |  |
| Yes | No | 2.51(1.92, 3.29) |  |  |  |
| No | Yes | 3.79(1.82, 7.86) |  |  |  |
| Yes | Yes | 8.61(4.28, 17.31) |  |  |  |
| Social support | Anxiety symptoms |  | 2.96(1.02, 4.90) | 0.46(0.38, 0.54) | 2.20(1.65, 2.93) |
| High | No | 1.00 |  |  |  |
| Low | No | 1.61(1.10, 2.35) |  |  |  |
| High | Yes | 2.87(1.38, 5.95) |  |  |  |
| Low | Yes | 6.43(3.22, 12.86) |  |  |  |

AOR, adjusted odds ratio; RERI, the relative excess risk due to interaction; AP, the attributable proportion due to interaction; S, the synergy index.

†Adjusted for gender, education, marital status, smoking, alcohol drinking, physical exercise, descendants, medical insurance, monthly individual income, length of stay, depression symptoms and ADL status, as well as the variables in the table.

**Discussion**

In this study, we examined the prevalence of poor sleep quality and its risk factors among elderly adults in nursing homes in China. Additionally, we explored the interactions of risk factors for poor sleep quality. We found that poor sleep quality affected nearly two-thirds of elderly residents in nursing homes and that older age, lower education, chronic diseases, depression symptoms, anxiety symptoms and lower social support were risk factors for poor sleep quality. Moreover, additive interactions were detected between age and anxiety symptoms and between education and anxiety symptoms.

This large population-based study used a standardized and valid PSQI tool to measure the characteristics of sleep quality among elderly adults in nursing homes in China. Based on this approach, we found that the prevalence of poor sleep quality was 67.3% among elderly residents in nursing homes in China. This finding is comparable to those of other previous studies conducted in elderly adults living in nursing homes in Turkey[31, 32]. However, with a similar population, the same institutional setting and measurement tool, Stefan et al [33] found that 54.5% of individuals were poor sleepers among 894 elderly adults in Zagreb nursing homes. Tsai et al [34] found that 46.4% of participants reported poor sleep quality among 196 elderly nursing home residents in Taiwan. A reason for the variance in these estimates may be explained by the different inclusion criteria for the study sample, as well as the differences in facilities and medical care among different institutions. As mentioned in the introduction, some sleep problems may be exacerbated by institutional setting. Our study also indicates that the prevalence of poor sleep quality in nursing homes was higher than in community homes. For instance, only 33.8% of community-dwelling elderly adults in Yanggu County[8] and 27.7% in Deqing County[35] in China reported poor sleep quality. On the one hand, the high self-rated prevalence may result from residents in nursing homes being older (55.1% of participants were aged 80 years and above in this study) and having more chronic conditions (78.5% of participants have at least one kind of chronic disease) than elderly adults in the community[36]. On the other hand, an uncomfortable environment and care routines in the institution may not promote sleep[37]. Among the seven PSQI components, lower sleep efficiency (62.2%) was the most common, followed by longer sleep latency (40.3%) and sleep disturbances (30.4%). These results were consistent with those of previous studies [31, 32]. As shown in our study, elderly adults suffered from several negative emotions (e.g., depression and anxiety) that may lead to longer sleep latency and lower sleep efficiency[38]. The main cause of sleep disturbances in nursing home settings may be nocturia in elderly adults, as well as noise and an uncomfortable environment[39]. However, the use of sleep medication was not prevalent in this population, and only 14.1% of individuals reported that they had taken sleep medication in the previous month. These results indicated that sleep problems in residents may not attract enough attention by administrative and healthcare staff in nursing homes. Otherwise, people had no awareness and believed that they were obtaining healthcare services for sleep problems in such a setting.

Consistent with previous epidemiological studies in the community, our study found that the prevalence of poor sleep quality was higher in older participants [9, 40]. However, the findings of studies on the association between sleep quality and gender were conflicting. Many studies indicate that females are more prone than males to experience poor sleep quality because a higher proportion of females have lower socioeconomic status and are more susceptible to anxiety and depression[40, 41]. In contrast, some studies indicate that males are more likely to report sleep problems[42, 43]. In our study, no significant difference in prevalence was observed between males and females, which is consistent with the findings of a study conducted among elderly attendees of a primary care centre in Malaysia [44] and the findings of another study conducted in nursing homes in Taiwan[34]. The differences in these findings may be related to the differences in the study sample and research location. For other socio-demographic factors, a shorter education duration was positively associated with poor sleep quality after adjustments were made for other confounders. This finding is consistent with those of other previous studies in both institutionalized and non-institutionalized elderly individuals. One possible reason is that less educated elderly adults may have limited ability to obtain and utilize information to manage some sleep problems, thus contributing to poor sleep quality. Specific chronic conditions, such as hypertension[45], heart disease[44], arthritis[9] and COPD[8], were found to be associated with poor sleep quality. In our current study, one interesting finding is that the risk of poor sleep quality was increased with an increasing number of chronic diseases. This result is in accordance with those of other previous studies. For example, a cross-sectional study performed among 16,680 residents aged 65 years in eight low- and middle-income countries demonstrated that poor health and a high number of comorbidities were associated with more sleep complaints[46]. Another population-based cross-sectional study among 5,107 adults in Japan suggested that the PSQI score increased linearly as the number of comorbid conditions increased [47]. This phenomenon provides new insights into this association, which may be caused by some type of cluster effect between multiple chronic conditions and requires further investigation.

Consistent with the findings of previous studies, depression and anxiety independently increased the risk of poor sleep quality[48-50]. For example, Cho HJ et al[51] found that a greater level of depressive symptoms had increased odds of sleep latency ≥1 hour in 3,051 participants aged 67 and older in the US. Another study conducted among 2,040 elderly Koreans suggested that poor subjective sleep quality, longer sleep latency and the frequent use of sleeping medication were independently associated with depression[52]. Potvin and colleague [53] suggest that nearly all PSQI subscales were significantly associated with anxiety, especially those for daytime sleepiness and sleep disturbances. Press et al [54] reported that depressive symptoms were associated with decreased sleep satisfaction, while anxiety symptoms were associated with difficulty falling asleep, waking up during the night and morning weakness. In addition, we also discovered that anxiety symptoms not only is an independent risk factor for poor sleep quality but also has additive interactions with older age and chronic disease. In other words, anxious participants who are also older in age or who have a chronic disease would have a higher prevalence of poor sleep quality. Many studies have reported that anxiety symptoms are more commonly seen in older people[55, 56]. Additionally, studies have indicated that chronic disease and anxiety symptoms occur contemporaneously, which may reinforce each other and result in a much stronger association[57, 58]. These results may partly explain these interactions between three risk factors and provide new insights into the prevention and treatment of sleep problems.

We assessed social support using the SSRS, which is a self-rated questionnaire developed by Xiao and widely used in epidemiological studies in China. This perceived social support scale evaluates social relationships with friends, neighbours, colleagues and family members as well as the availability of functional support. Many studies consider social support to be an important factor in the maintenance of health status. In our study, we found that a lower level of social support increased the risk of poor sleep quality in nursing home residents. This result was consistent with the findings of previous studies[59-61]. For example, Kishimoto et al [60] found that individuals with weak social support from spouses or family members had significantly increased adjusted odds ratios for the risk of sleep disturbances. Another study[61] conducted among 998 African-American elderly adults indicated that those who reported lower levels of emotional social support have more difficulties in initiating and maintaining sleep. First, social support may influence sleep quality by enabling a feeling of belonging and protection from social isolation and negative emotions (e.g., loneliness, depression and anxiety), thus decreasing the incident risk of sleep disorders [62-64]. Similar to our study results, people with anxiety had an increased risk of poor sleep quality, especially those with lower social support. Additionally, a stronger social network may contribute to individuals having multiple sources of health information, thus increasing the probability of obtaining information that may help maintain and promote healthy sleep behaviour [65, 66].

To the best of our knowledge, this is the first study to explore the risk factors and their interactions for poor sleep quality among elderly adults in nursing homes in China. With a large population-based sample and a higher response rate, these findings of our study may provide valuable information for sleep among aging populations in nursing homes. From a clinical point of view, concern about mental health in residents and intervention and treatment for anxiety symptoms may be helpful in the improvement of sleep quality in nursing homes. Additionally, the amelioration of the living environment and the provision of more social support were also important. However, there are also several limitations that need to be addressed. First, the causation between some risk factors and poor sleep quality cannot be inferred due to the cross-sectional study design. For example, some studies also declared that poor sleep quality was positively associated with depression, anxiety and chronic diseases[67, 68]. Second, all data were obtained through self-rating and may introduce recall bias. Third, the limited sensitivity and specificity of the Chinese version of PSQI might lead to misclassifications for poor sleepers. Finally, our study only provided evidence on these associations among elderly adults in nursing homes. Therefore, these limitations need to be fully considered when generalizing these results to community-dwelling elderly adults.

**Conclusions**

The prevalence of poor sleep quality in nursing homes is relatively high. The risk factors for poor sleep quality were older age, lower education, chronic diseases, depression symptoms, anxiety symptoms and lower social support. Anxiety symptoms has additive interactions with age, chronic disease and social support for poor sleep quality. These findings have significant implications for interventions that aim to improve sleep quality among elderly residents in nursing homes.

**Acknowledgements**

We thank all elderly residents in nursing homes for their cooperation.

**References**

1.	National Bureau of Statistics of China. The Sixth National Population Census Data Gazette (No. 1). National Census Bureau. Beijing, China: 2011.

2.	National committee on aging.Report on prediction of population aging trend in China. <http://www.cncaprc.gov.cn/contents/16/11224.html>.; 2007-12-27.

3.	Maggi S, Langlois JA, Minicuci N, Grigoletto F, Pavan M, Foley DJ, et al. Sleep complaints in community-dwelling older persons: prevalence, associated factors, and reported causes. J Am Geriatr Soc. 1998;46(2):161-8. Epub 1998/02/25. PubMed PMID: 9475443.

4.	Roepke SK, Ancoli-Israel S. Sleep disorders in the elderly. The Indian journal of medical research. 2010;131:302-10. Epub 2010/03/24. PubMed PMID: 20308755.

5.	Roberts RE, Shema SJ, Kaplan GA, Strawbridge WJ. Sleep complaints and depression in an aging cohort: A prospective perspective. The American journal of psychiatry. 2000;157(1):81-8. Epub 2000/01/05. doi: 10.1176/ajp.157.1.81. PubMed PMID: 10618017.

6.	Aunjitsakul W, Pitanupong J, Werachattawan N. Sleep Quality among Elderly People in Songkhla Province, Thailand: A Two-Stage Cluster Sampling Study. Journal of the Medical Association of Thailand. 2018;101(1):137-44. PubMed PMID: BCI:BCI201800464697.

7.	Pedraza S, Al Snih S, Ottenbacher KJ, Markides KS, Raji MA. Sleep quality and sleep problems in Mexican Americans aged 75 and older. Aging Clin Exp Res. 2012;24(4):391-7. Epub 2011/11/22. doi: 10.3275/8106. PubMed PMID: 22102513; PubMed Central PMCID: PMCPMC3521094.

8.	Wang P, Song L, Wang K, Han X, Cong L, Wang Y, et al. Prevalence and associated factors of poor sleep quality among Chinese older adults living in a rural area: a population-based study. Aging clinical and experimental research. 2019. doi: 10.1007/s40520-019-01171-0. PubMed PMID: MEDLINE:30919262.

9.	Luo J, Zhu G, Zhao Q, Guo Q, Meng H, Hong Z, et al. Prevalence and Risk Factors of Poor Sleep Quality among Chinese Elderly in an Urban Community: Results from the Shanghai Aging Study. Plos One. 2013;8(11). doi: 10.1371/journal.pone.0081261. PubMed PMID: WOS:000327543500078.

10.	Martins da Silva R, Afonso P, Fonseca M, Teodoro T. Comparing sleep quality in institutionalized and non-institutionalized elderly individuals. Aging & mental health. 2019:1-7. Epub 2019/05/18. doi: 10.1080/13607863.2019.1619168. PubMed PMID: 31099258.

11.	Fetveit A, Bjorvatn B. Sleep disturbances among nursing home residents. International journal of geriatric psychiatry. 2002;17(7):604-9. doi: 10.1002/gps.639. PubMed PMID: WOS:000176887000002.

12.	Uchmanowicz I, Markiewicz K, Uchmanowicz B, Koltuniuk A, Rosinczuk J. The relationship between sleep disturbances and quality of life in elderly patients with hypertension. Clinical interventions in aging. 2019;14:155-65. Epub 2019/01/31. doi: 10.2147/cia.s188499. PubMed PMID: 30697040; PubMed Central PMCID: PMCPMC6339653.

13.	Zhang H, Li Y, Zhao X, Mao Z, Abdulai T, Liu X, et al. The association between PSQI score and hypertension in a Chinese rural population: the Henan Rural Cohort Study. Sleep medicine. 2019;58:27-34. Epub 2019/05/07. doi: 10.1016/j.sleep.2019.03.001. PubMed PMID: 31059971.

14.	Hoevenaar-Blom MP, Spijkerman AM, Kromhout D, van den Berg JF, Verschuren WM. Sleep duration and sleep quality in relation to 12-year cardiovascular disease incidence: the MORGEN study. Sleep. 2011;34(11):1487-92. Epub 2011/11/02. doi: 10.5665/sleep.1382. PubMed PMID: 22043119; PubMed Central PMCID: PMCPMC3198203.

15.	Gelaye B, Okeiga J, Ayantoye I, Berhane HY, Berhane Y, Williams MA. Association of suicidal ideation with poor sleep quality among Ethiopian adults. Sleep & breathing = Schlaf & Atmung. 2016;20(4):1319-26. Epub 2016/10/25. doi: 10.1007/s11325-016-1418-9. PubMed PMID: 27771845; PubMed Central PMCID: PMCPMC5159269.

16.	Livingston G, Blizard B, Mann A. Does sleep disturbance predict depression in elderly people? A study in inner London. The British journal of general practice : the journal of the Royal College of General Practitioners. 1993;43(376):445-8. Epub 1993/11/01. PubMed PMID: 8292414; PubMed Central PMCID: PMCPMC1372481.

17.	Sukegawa T, Itoga M, Seno H, Miura S, Inagaki T, Saito W, et al. Sleep disturbances and depression in the elderly in Japan. Psychiatry Clin Neurosci. 2003;57(3):265-70. doi: 10.1046/j.1440-1819.2003.01115.x. PubMed PMID: WOS:000182651600004.

18.	Stranges S, Tigbe W, Gomez-Olive FX, Thorogood M, Kandala NB. Sleep problems: an emerging global epidemic? Findings from the INDEPTH WHO-SAGE study among more than 40,000 older adults from 8 countries across Africa and Asia. Sleep. 2012;35(8):1173-81. Epub 2012/08/02. doi: 10.5665/sleep.2012. PubMed PMID: 22851813; PubMed Central PMCID: PMCPMC3397790.

19.	Thichumpa W, Howteerakul N, Suwannapong N, Tantrakul V. Sleep quality and associated factors among the elderly living in rural Chiang Rai, northern Thailand. Epidemiol Health. 2018;40:e2018018. Epub 2018/05/29. doi: 10.4178/epih.e2018018. PubMed PMID: 29807410; PubMed Central PMCID: PMCPMC6060346.

20.	Sagayadevan V, Abdin E, Binte Shafie S, Jeyagurunathan A, Sambasivam R, Zhang Y, et al. Prevalence and correlates of sleep problems among elderly Singaporeans. Psychogeriatrics. 2017;17(1):43-51. doi: 10.1111/psyg.12190. PubMed PMID: WOS:000393967000006.

21.	de Pooter-Stijnman LMM, Vrijkotte S, Smalbrugge M. Effect of caffeine on sleep and behaviour in nursing home residents with dementia. European geriatric medicine. 2018;9(6):829-35. Epub 2018/12/24. doi: 10.1007/s41999-018-0115-6. PubMed PMID: 30574215; PubMed Central PMCID: PMCPMC6267648.

22.	Quan SA, Li YC, Li WJ, Li Y, Jeong JY, Kim DH. Gender Differences in Sleep Disturbance among Elderly Koreans: Hallym Aging Study. Journal of Korean medical science. 2016;31(11):1689-95. Epub 2016/10/07. doi: 10.3346/jkms.2016.31.11.1689. PubMed PMID: 27709844; PubMed Central PMCID: PMCPMC5056198.

23.	Su TP, Huang SR, Chou P. Prevalence and risk factors of insomnia in community-dwelling Chinese elderly: a Taiwanese urban area survey. The Australian and New Zealand journal of psychiatry. 2004;38(9):706-13. Epub 2004/08/25. doi: 10.1080/j.1440-1614.2004.01444.x. PubMed PMID: 15324335.

24.	Lawton MP, Brody EM. Assessment of older people: self-maintaining and instrumental activities of daily living. The Gerontologist. 1969;9(3):179-86. doi: 10.1093/geront/9.3_Part_1.179. PubMed PMID: MEDLINE:5349366.

25.	Smalbrugge M, Jongenelis L, Pot AM, Beckman ATF, Eefsting JA. Screening for depression and assessing change in severity of depression. Is the Geriatric Depression Scale (30-, 15- and 8-item versions) useful for both purposes in nursing home patients? Aging & mental health. 2008;12(2):244-8. doi: 10.1080/13607860801987238. PubMed PMID: WOS:000255342900011.

26.	Lu J, Wang Y, Wang X, Song R, Yi X. Reliability and Validity of the Chinese Version of Geriatric Depression Scale Among Chinese Urban Community-dwelling Elderly Population. Chinese Journal of Clinical Psychology. 2013;21(1):39-41.

27.	Spitzer RL, Kroenke K, Williams JBW, Loewe B. A brief measure for assessing generalized anxiety disorder - The GAD-7. Archives of Internal Medicine. 2006;166(10):1092-7. doi: 10.1001/archinte.166.10.1092. PubMed PMID: WOS:000237720500008.

28.	Buysse DJ, Reynolds CF, 3rd, Monk TH, Berman SR, Kupfer DJ. The Pittsburgh Sleep Quality Index: a new instrument for psychiatric practice and research. Psychiatry research. 1989;28(2):193-213. doi: 10.1016/0165-1781(89)90047-4. PubMed PMID: MEDLINE:2748771.

29.	Jian-qing Q, Mi Z, Pei-yuan Q, Na Z, Yang W. Reliability and Validity of the Pittsburgh Sleep Quality Index in older adults in rural area. Modern Preventive Medicine. 2016;43(10):1835-8.In Chinese.

30.	Andersson T, Alfredsson L, Kallberg H, Zdravkovic S, Ahlbom A. Calculating measures of biological interaction. European journal of epidemiology. 2005;20(7):575-9. Epub 2005/08/27. PubMed PMID: 16119429.

31.	Orhan FO, Tuncel D, Tas F, Demirci N, Ozer A, Karaaslan MF. Relationship between sleep quality and depression among elderly nursing home residents in Turkey. Sleep and Breathing. 2012;16(4):1059-67. doi: 10.1007/s11325-011-0601-2. PubMed PMID: WOS:000311301700020.

32.	Eser I, Khorshid L, Cinar S. Sleep quality of older adults in nursing homes in Turkey: enhancing the quality of sleep improves quality of life. Journal of gerontological nursing. 2007;33(10):42-9. Epub 2007/10/25. PubMed PMID: 17955737.

33.	Stefan L, Vrgoc G, Rupcic T, Sporis G, Sekulic D. Sleep Duration and Sleep Quality Are Associated with Physical Activity in Elderly People Living in Nursing Homes. International journal of environmental research and public health. 2018;15(11). Epub 2018/11/15. doi: 10.3390/ijerph15112512. PubMed PMID: 30423981; PubMed Central PMCID: PMCPMC6266288.

34.	Tsai YF, Wong TKS, Ku YC. Self-care management of sleep disturbances and risk factors for poor sleep among older residents of Taiwanese nursing homes. Journal of clinical nursing. 2008;17(9):1219-26. doi: 10.1111/j.1365-2702.2007.02020.x. PubMed PMID: WOS:000254990600011.

35.	Dong X, Wang Y, Chen Y, Wang X, Zhu J, Wang N, et al. Poor sleep quality and influencing factors among rural adults in Deqing, China. Sleep & breathing = Schlaf & Atmung. 2018;22(4):1213-20. Epub 2018/06/25. doi: 10.1007/s11325-018-1685-8. PubMed PMID: 29936592.

36.	Gnadinger M, Herzig L, Ceschi A, Conen D, Staehelin A, Zoller M, et al. Chronic conditions and multimorbidity in a primary care population: a study in the Swiss Sentinel Surveillance Network (Sentinella). International journal of public health. 2018;63(9):1017-26. Epub 2018/05/23. doi: 10.1007/s00038-018-1114-6. PubMed PMID: 29786762; PubMed Central PMCID: PMCPMC6245242.

37.	Gentili A, Weiner DK, Kuchibhatil M, Edinger JD. Factors that disturb sleep in nursing home residents. Aging (Milan, Italy). 1997;9(3):207-13. doi: 10.1007/bf03340151. PubMed PMID: MEDLINE:9258380.

38.	Gentili A, Weiner DK, Kuchibhatil M, Edinger JD. Factors that disturb sleep in nursing home residents. Aging (Milano). 1997;9(3):207-13. Epub 1997/06/01. PubMed PMID: 9258380.

39.	Ersser S, Wiles A, Taylor H, Wade S, Walsh R, Bentley T. The sleep of older people in hospital and nursing homes. Journal of clinical nursing. 1999;8(4):360-8. Epub 2000/01/07. PubMed PMID: 10624252.

40.	Wang Y, Li Y, Liu X, Liu R, Mao Z, Tu R, et al. Gender-specific prevalence of poor sleep quality and related factors in a Chinese rural population: the Henan Rural Cohort Study. Sleep medicine. 2019;54:134-41. Epub 2018/12/17. doi: 10.1016/j.sleep.2018.10.031. PubMed PMID: 30554057.

41.	Kara B, Tenekeci E. Sleep Quality and Associated Factors in Older Turkish Adults With Hypertension: A Pilot Study. J Transcult Nurs. 2017;28(3):296-305. doi: 10.1177/1043659615623330. PubMed PMID: WOS:000403944700010.

42.	Wilfling D, Dichter MN, Trutschel D, Kopke S. Prevalence of Sleep Disturbances in German Nursing Home Residents with Dementia: A Multicenter Cross-Sectional Study. Journal of Alzheimer's disease : JAD. 2019;69(1):227-36. Epub 2019/04/09. doi: 10.3233/jad-180784. PubMed PMID: 30958349.

43.	Gu DN, Sautter J, Pipkin R, Zeng Y. Sociodemographic and Health Correlates of Sleep Quality and Duration among Very Old Chinese. Sleep. 2010;33(5):601-10. doi: 10.1093/sleep/33.5.601. PubMed PMID: WOS:000277232200007.

44.	Razali R, Ariffin J, Aziz AFA, Puteh SEW, Wahab S, Daud TIM. Sleep quality and psychosocial correlates among elderly attendees of an urban primary care centre in Malaysia. Neurol Asia. 2016;21(3):265-73. PubMed PMID: WOS:000385421900008.

45.	Yang CY, Chiou AF. Predictors of Sleep Quality in Community-Dwelling Older Adults in Northern Taiwan. J Nurs Res. 2012;20(4):249-60. doi: 10.1097/jnr.0b013e3182736461. PubMed PMID: WOS:000311276900003.

46.	Mazzotti DR, Guindalini C, Sosa AL, Ferri CP, Tufik S. Prevalence and correlates for sleep complaints in older adults in low and middle income countries: A 10/66 Dementia Research Group study. Sleep medicine. 2012;13(6):697-702. doi: 10.1016/j.sleep.2012.02.009. PubMed PMID: WOS:000306304700024.

47.	Hayashino Y, Yamazaki S, Takegami M, Nakayama T, Sokejima S, Fukuhara S. Association between number of comorbid conditions, depression, and sleep quality using the Pittsburgh Sleep Quality Index: results from a population-based survey. Sleep medicine. 2010;11(4):366-71. Epub 2010/03/12. doi: 10.1016/j.sleep.2009.05.021. PubMed PMID: 20219425.

48.	Park JH, Yoo MS, Bae SH. Prevalence and predictors of poor sleep quality in Korean older adults. Int J Nurs Pract. 2013;19(2):116-23. doi: 10.1111/ijn.12047. PubMed PMID: WOS:000317675800002.

49.	Yu J, Rawtaer I, Fam J, Jiang MJ, Feng L, Kua EH, et al. Sleep correlates of depression and anxiety in an elderly Asian population. Psychogeriatrics. 2016;16(3):191-5. Epub 2015/07/17. doi: 10.1111/psyg.12138. PubMed PMID: 26179204.

50.	Paudel ML, Taylor BC, Diem SJ, Stone KL, Ancoli-Israel S, Redline S, et al. Association between depressive symptoms and sleep disturbances in community-dwelling older men. Journal of the American Geriatrics Society. 2008;56(7):1228-35. doi: 10.1111/j.1532-5415.2008.01753.x. PubMed PMID: WOS:000258073800009.

51.	Cho HJ, Lavretsky H, Olmstead R, Levin MJ, Oxman MN, Irwin MR. Sleep Disturbance and Depression Recurrence in Community-Dwelling Older Adults: A Prospective Study. American Journal of Psychiatry. 2008;165(12):1543-50. doi: 10.1176/appi.ajp.2008.07121882. PubMed PMID: WOS:000261266000011.

52.	Chang KJ, Son SJ, Lee Y, Back JH, Lee KS, Lee SJ, et al. Perceived sleep quality is associated with depression in a Korean elderly population. Archives of Gerontology and Geriatrics. 2014;59(2):468-73. doi: 10.1016/j.archger.2014.04.007. PubMed PMID: WOS:000341018300038.

53.	Potvin O, Lorrain D, Belleville G, Grenier S, Preville M. Subjective sleep characteristics associated with anxiety and depression in older adults: a population-based study. International journal of geriatric psychiatry. 2014;29(12):1262-70. doi: 10.1002/gps.4106. PubMed PMID: WOS:000345214600010.

54.	Press Y, Punchik B, Freud T. The association between subjectively impaired sleep and symptoms of depression and anxiety in a frail elderly population. Aging Clinical and Experimental Research. 2018;30(7):755-65. doi: 10.1007/s40520-017-0837-1. PubMed PMID: WOS:000435713200007.

55.	Ahmed D, El Shair IH, Taher E, Zyada F. Prevalence and predictors of depression and anxiety among the elderly population living in geriatric homes in Cairo, Egypt. The Journal of the Egyptian Public Health Association. 2014;89(3):127-35. doi: 10.1097/01.epx.0000455729.66131.49. PubMed PMID: MEDLINE:25534177.

56.	Kirmizioglu Y, Dogan O, Kugu N, Akyuz G. Prevalence of anxiety disorders among elderly people. International journal of geriatric psychiatry. 2009;24(9):1026-33. doi: 10.1002/gps.2215. PubMed PMID: WOS:000269428100017.

57.	Teesson M, Mitchell PB, Deady M, Memedovic S, Slade T, Baillie A. Affective and anxiety disorders and their relationship with chronic physical conditions in Australia: findings of the 2007 National Survey of Mental Health and Wellbeing. Aust N Z J Psych. 2011;45(11):939-46. doi: 10.3109/00048674.2011.614590. PubMed PMID: WOS:000296457400007.

58.	Beekman ATF, de Beurs E, van Balkom A, Deeg DJH, van Dyck R, van Tilburg W. Anxiety and depression in later life: Co-occurrence and communality of risk factors. American Journal of Psychiatry. 2000;157(1):89-95. doi: 10.1176/ajp.157.1.89. PubMed PMID: WOS:000084559900014.

59.	Eshkoor SA, Hamid TA, Nudin SSH, Mun CY. The Effects of Social Support and Having a Partner on Sleep Quality in Dementia. Am J Alzheimers Dis Other Dement. 2013;28(3):253-7. doi: 10.1177/1533317513481098. PubMed PMID: WOS:000318525500007.

60.	Kishimoto Y, Okamoto N, Saeki K, Tomioka K, Obayashi K, Komatsu M, et al. Bodily pain, social support, depression symptoms and stroke history are independently associated with sleep disturbance among the elderly: a cross-sectional analysis of the Fujiwara-kyo study. Environ Health Prev. 2016;21(5):295-303. doi: 10.1007/s12199-016-0529-z. PubMed PMID: WOS:000381290400002.

61.	Bazargan M. Self-reported sleep disturbance among African-American elderly: the effects of depression, health status, exercise, and social support. International journal of aging & human development. 1996;42(2):143-60. doi: 10.2190/gm89-nrty-derq-lc7d. PubMed PMID: MEDLINE:8859496.

62.	Troxel WM, Buysse DJ, Monk TH, Begley A, Hall M. Does social support differentially affect sleep in older adults with versus without insomnia? Journal of psychosomatic research. 2010;69(5):459-66. Epub 2010/10/20. doi: 10.1016/j.jpsychores.2010.04.003. PubMed PMID: 20955865; PubMed Central PMCID: PMCPMC2958100.

63.	Olivera J, Benabarre S, Lorente T, Rodriguez M, Peleogrin C, Calvo JM, et al. Prevalence of psychiatric symptoms and mental disorders detected in primary care in an elderly Spanish population. The PSICOTARD Study: preliminary findings. International journal of geriatric psychiatry. 2008;23(9):915-21. doi: 10.1002/gps.2004. PubMed PMID: WOS:000259148300006.

64.	Paukert AL, Pettit JW, Kunik ME, Wilson N, Novy DM, Rhoades HM, et al. The Roles of Social Support and Self-Efficacy in Physical Health's Impact on Depressive and Anxiety Symptoms in Older Adults. J Clin Psychol Med Settings. 2010;17(4):387-400. doi: 10.1007/s10880-010-9211-6. PubMed PMID: WOS:000285154000013.

65.	Cacioppo JT, Hawkley LC. Social isolation and health, with an emphasis on underlying mechanisms. Perspectives in biology and medicine. 2003;46(3 Suppl):S39-52. Epub 2003/10/18. PubMed PMID: 14563073.

66.	Costa SV, Ceolim MF, Neri AL. Sleep problems and social support: Frailty in a Brazilian Elderly Multicenter study. Revista latino-americana de enfermagem. 2011;19(4):920-7. Epub 2011/08/31. doi: 10.1590/s0104-11692011000400010. PubMed PMID: 21876944.

67.	Matsuda R, Kohno T, Kohsaka S, Fukuoka R, Maekawa Y, Sano M, et al. The prevalence of poor sleep quality and its association with depression and anxiety scores in patients admitted for cardiovascular disease: A cross-sectional designed study. Int J Cardiol. 2017;228:977-82. Epub 2016/12/05. doi: 10.1016/j.ijcard.2016.11.091. PubMed PMID: 27915216.

68.	Bo Y, Yeoh EK, Guo C, Zhang Z, Tam T, Chan TC, et al. Sleep and the Risk of Chronic Kidney Disease: A Cohort Study. Journal of clinical sleep medicine : JCSM : official publication of the American Academy of Sleep Medicine. 2019;15(3):393-400. Epub 2019/03/12. doi: 10.5664/jcsm.7660. PubMed PMID: 30853043; PubMed Central PMCID: PMCPMC6411180.
