# Supplementary material for: The prevalence of poor sleep quality and associated risk factors among Chinese elderly adults in nursing homes: A cross-sectional study
Source: PLoS One. 2020 May 15;15(5):e0232834. doi: 10.1371/journal.pone.0232834 (PMC7228093; doi:10.1371/journal.pone.0232834)
Supplement: Attachment — Submitted filename: Response to Reviewers.docx [file pone.0232834.s004.docx]

Response to [PONE-D-19-31759R1]

Thank you very much for your valuable comments and critiques, which have improved the clarity of our manuscript to a greater extent. Accordingly, we have thoroughly revised our manuscript based on the editors’ and reviewers’ comments and suggestions. The changes are highlighted in red for added words, or strikethrough for deleted words. Additionally, minor language, grammatical and stylistic errors have been corrected. Our specific point-by-point responses to the comments and queries have been addressed and itemized as follows:

Reviewer #1: this study is good enough but it may need some improvement data such as more specific characteristic demography, analysis data, and explanation more about quality of life among Chinese elderly adults.

**Answer:** Thank you so much for your valuable comment. As suggested we have included and described more specific socio-demographic characteristics of the sample in relation to the study objectives. We did not dwell much on discussing quality of life of the elderly in the nursing homes because our main focus was on sleep quality, a component of quality life, and its associated factors. Thus, we justified why it was important to study this phenomenon. However, we agree that an explanation about the quality of life among the Chinese elderly people would highlight some insights in understanding some issues that would affect the living conditions of this population. Therefore, we have added a paragraph in the section of introduction to describe some facts about the quality of life of this group of people. Nevertheless, this study did not examine the quality of life of this population in general, but just a component of it, namely sleep quality. We thank you so much for your observation, which we have taken as a consideration to investigate the quality of life of this population in our future research.

Reviewer #3: This is a cross-sectional study examining in 817 elderly residents in nursing homes the prevalence of poor sleep quality and its risk factors
The study confirms a relatively high prevalence of poor sleep quality in nursing homes, previously reported. Anxiety symptoms had additive interactions with age, chronic disease and social support for poor sleep quality.

On the whole, I have a quite positive opinion on this cross-sectional study. It has a quite large sample size and methods seem mostly accurate.

Major concerns:
1. I understand but not completely agree on the reduction of scores of anxiety, depression and social support to categorical variables, using a cut off. I ask to integrate the current analyses using the actual scores. Not necessarily, this request means a deletion of categorical classifications, but continuous variables should also be consider din the analyses

**Answer:** Thank you so much for your valuable comment. It should be noted that the tools that we used to measure anxiety symptoms, depression symptoms and social support have high validity and reliability, and they are widely used to investigate anxiety, depression and social support among the Chinese elderly people. Besides, the cut-off points that we used on the total scores of these tools were adopted from other published studies on similar populations. However, according to the reviewer’s suggestion, we tried to consider anxiety, depression and social support as continuous variables, but the results did not deviate much from those when anxiety, depression and social support were considered as categorical variables (see the Table 1 below). Essentially, the results in the Table 1 below mean pretty much the same thing as the results in our original analysis .

Table 1 Risk factors associated with poor sleep quality among the elderly in nursing homes

| Variable | B | SE | Wald | AOR(95% CI)^†^ | P-value |
| --- | --- | --- | --- | --- | --- |
| Age (years) |  |  |  |  |  |
| 60~ |  |  |  | 1.00 |  |
| 70~ | 0.58 | 0.25 | 5.15 | 1.78(1.08,2.92) | 0.023 |
| 80~ | 0.98 | 0.24 | 17.37 | 2.67(1.68,4.24) | <0.001 |
| Number of chronic diseases |  |  |  |  |  |
| 0~ |  |  |  |  |  |
| 1~ | 0.72 | 0.20 | 13.31 | 2.05(1.39,3.01) | <0.001 |
| 3~ | 0.86 | 0.27 | 10.01 | 2.35(1.39,4.00) | 0.002 |
| Depression symptoms | 0.07 | 0.02 | 17.29 | 1.08(1.04,1.11) | <0.001 |
| Anxiety symptoms | 0.11 | 0.03 | 14.02 | 1.11(1.05,1.18) | <0.001 |
| Social support | -0.03 | 0.02 | 5.32 | 0.97(0.95,0.99) | 0.021 |

2. Due to the number of comparisons performed, a significance level set al p<0.05 should be adequately corrected for multiple comparisons

**Answer:** Thank you so much for your valuable comment. A p<0.025 was set to indicate significant results for multiple comparisons in this study.

3. Due to the potential effects of illumination levels in nursing homes on sleep-wake activity rhythms (e.g., Ancoli‐Israel et al. , 9: 373-379, 2000). I ask to collect (if possible) this information in the considered nursing homes and include this variable in their analyses

**Answer:** Thank you so much for your valuable comment. We have not collected this information in our investigation; therefore we will consider this information in our further study.
